# CD20 expression dynamics in adult B-cell acute lymphoblastic leukemia and impact on anti-CD20 treatment

**DOI:** 10.1186/s13045-026-01832-4

**Published:** 2026-07-20

**Authors:** Monika Szczepanowski, Johanna Richter, Sonja Bendig, Lorenz Bastian, Miriam Kelm, Veronika Beck, Thomas Beder, Guranda Chitadze, Michaela Kotrová, Johannes Duell, Christoph Faul, Johannes Gärtner, Alina Hartmann, Britta Kehden, Eva Maria Murga Penas, Martin Neumann, Matthias Ritgen, Wiebke Schrader, Björn Steffen, Heiko Trautmann, Andreas Viardot, Thomas Burmeister, Claudia D. Baldus, Stefan Schwartz, Nicola Gökbuget, Monika Brüggemann

**Affiliations:** 1 Medicine Department II, Hematology and Oncology, University Medical Center Schleswig-Holstein (UKSH), Campus Kiel, Kiel, Germany; 2 Department of Internal Medicine II, Würzburg University Hospital, University of Würzburg, Würzburg, Germany; 3 Internal Medicine II, University Hospital and Comprehensive Cancer Center Tübingen, Universitätsklinikum Tübingen, Tübingen, Germany; 4 Department of Medicine 5, Oncology, Hematology and Bone Marrow Transplantation Unit, Klinikum Nürnberg Nord, Nürnberg, Germany; 5 Institute of Human Genetics, University Medical Center Schleswig- Holstein (UKSH), Campus Lübeck, Lübeck, Germany; 6 Medicine Department II, Johann Wolfgang Goethe University, Frankfurt, Germany; 7 Department of Internal Medicine, University Hospital of Ulm, Ulm, Germany; 8 Department of Hematology, Oncology and Tumor Immunology, Campus Virchow Klinikum, Campus Virchow Klinikum, Charité - Universitätsmedizin, Berlin, Germany; 9 Corporate member of Freie Universität Berlin and Humboldt-Universität zu Berlin , Berlin, Germany; 10 Department of Hematology, Oncology and Tumor Immunology, Campus Benjamin Franklin, Charité - Universitätsmedizin Berlin, Berlin, Germany; 11 Charité - Universitätsmedizin Berlin, corporate member of Freie Universität Berlin and Humboldt-Universität zu Berlin, Berlin, Germany; 12 German Cancer Consortium (DKTK) and German Cancer Research Center (DKFZ), Heidelberg, Germany; 13 Department for Stem Cell Transplantation, University Medical Center Hamburg-Eppendorf, Hamburg, Germany; 14 Clinical Research Unit ’CATCH ALL’ (KFO 5010), Kiel, Germany; 15 University Cancer Center Schleswig-Holstein (UCCSH), Campus Kiel, Kiel, Germany

**Keywords:** B-ALL, CD20 upregulation, Rituximab, Measurable residual disease, Early treatment response

## Abstract

**Background:**

Baseline CD20 expression ≥ 20% in B-cell acute lymphoblastic leukemia (B-ALL) has been associated with poorer outcomes, which improved after rituximab introduction into frontline therapy for CD20-positive cases. We investigated the clinical and biological significance of this threshold by monitoring early dynamics of CD20 expression.

**Methods:**

In the GMALL 08/2013 trial, adults with B-ALL received a cyclophosphamide/dexamethasone prephase, followed by Induction and Consolidation I, which included four rituximab doses in *BCR::ABL1*-negative patients, irrespective of CD20 status. CD20 expression was measured by standardized multiparametric flow cytometry in 274 patients in bone marrow and blood at diagnosis and in blood after prephase. IG/TR based measurable residual disease (MRD) was correlated with baseline bone marrow or the highest CD20-positive blast percentage recorded throughout prephase. The historical GMALL 07/2003 cohort treated without rituximab served for comparison.

**Results:**

Baseline CD20 expression was significantly higher in blood than in bone marrow and increased further during prephase. In paired baseline samples, 6/76 c-/pre-B ALL cases (7.9%) were CD20-negative by bone marrow (< 20%) but positive in blood. In paired blood samples, 14/106 patients crossed the 20% threshold after prephase (12/86 c-/pre-B ALL, 13.9% and 2/20 pro-B ALL, 10.0%). Among 182 *BCR::ABL1*-negative patients, highest recorded CD20 across all time points classified 76 (41.8%) as < 20% and 106 (58.2%) as ≥ 20%. Higher CD20 expression was associated with improved MRD response under rituximab. Among MRD-evaluable patients after Induction I (*n* = 161), molecular complete remission (MolCR) was achieved in 10.6% of patients with CD20 expression < 20% compared with 30.5% of those with CD20 expression ≥ 20%. After Consolidation I (*n* = 159), corresponding MolCR rates were 50.0% and 72.6%, respectively. Associations were weaker when only baseline CD20 bone marrow status was considered. No association with MRD response was observed in GMALL 07/2003 patients treated without rituximab, suggesting a treatment-driven effect in GMALL 08/2013.

**Conclusions:**

CD20 expression in adult B-ALL varies from diagnostic bone marrow to blood and post-prephase measurements. The highest recorded CD20% value better predicts early MRD responses under rituximab. Post-prephase CD20 reassessment in blood identifies additional patients with eligibility for rituximab.

**Registry:**

ClinicalTrials.gov, TRN: NCT02881086 (2016-08-23); NCT00198991 (2005-09-12).

**Supplementary Information:**

The online version contains supplementary material available at 10.1186/s13045-026-01832-4.

## Background

The MS4A transmembrane protein family member CD20 is expressed in 30–40% of B-cell acute lymphoblastic leukemia (B-ALL) and was first targeted in high- and low-grade non-Hodgkin B-cell lymphoma studies, in which the introduction of rituximab, a therapeutic CD20-directed monoclonal antibody, into frontline therapies significantly improved overall survival [1–3]. This observation provided the rationale for testing rituximab in CD20-positive B-ALL. In the pre-rituximab era, baseline CD20 expression ≥ 20% on B-ALL blasts was associated with higher relapse rates and poorer outcomes [4], attaining its status as an adverse prognostic factor, even though some studies suggested CD20 expression alone lacks prognostic significance in *BCR::ABL1*-negative B-ALL [5]. The introduction of rituximab required practical eligibility criteria. Consequently, a threshold of ≥ 20% CD20-positive blasts at baseline was introduced to define CD20-positive B-ALL. Since then, B-ALL with CD20 expression below this threshold is classified as CD20-negative and considered ineligible for rituximab under current standard protocols. In the GRAALL-2005 study, adding rituximab to standard chemotherapy in de novo CD20-positive B-ALL improved complete remission (CR), overall survival (OS), and event-free survival (EFS) under standard chemotherapy regimens supplemented with either 8 or 16–18 doses of rituximab. Improved molecular CR rates and outcomes in standard-risk (SR) and high-risk (HR) patients with *BCR::ABL1*-negative CD20-positive B-ALL receiving 8 doses of rituximab were also reported in the German Multicenter Study Group for Adult ALL (GMALL) 07/2003 trial [6–10]. Conversely, in the UKALL14 trial, 4 rituximab doses added to induction chemotherapy (regardless of baseline CD20 or *BCR::ABL1* status) did not significantly improve EFS, although relapse rates were reduced, suggesting that extended dosing may be required [11].

In practice, determining optimal CD20 thresholds for rituximab eligibility and rituximab schedules is challenging due to the lack of a standardized CD20 assessment and heterogeneous trial designs, including differences in patient age, *BCR::ABL1* status, rituximab dosing number and time points, and varying applications of the 20% threshold. Consequently, robust comparative data on therapy response under rituximab, particularly for patients with low baseline CD20 expression, remain limited.

Additionally, CD20 expression can increase during early B-ALL treatment, as reported in pediatric studies in which prednisolone-induced CD20 upregulation conferred a higher sensitivity of blasts to rituximab-mediated cytotoxicity in vitro [12, 13]. Yet it remains unclear whether similar CD20 modulation occurs in adult B-ALL, whether it can be induced by dexamethasone, and whether CD20 expression levels are affected by microenvironmental factors in different body compartments (e.g., bone marrow (BM) and peripheral blood (PB)).

Current European LeukemiaNet (ELN) recommendations emphasize integrating morphology, multiparameter flow cytometry (FCM), and molecular genetics for accurate diagnosis, risk stratification, and effective measurable residual disease (MRD) monitoring. FCM is essential for determining lineage, maturation stage, and leukemia subtype, and for identifying immunophenotypic aberrancies relevant to FCM-based MRD detection, particularly in patients lacking suitable immunoglobulin and T-cell receptor gene rearrangement (IG/TR)-derived molecular markers. It also enables the identification of antigen targets for immune-based therapies [14].

Accurate quantification of baseline CD20 expression is crucial for the determination of rituximab eligibility in standard therapy settings, but guidelines for standardized CD20 assessment are currently lacking. Typically, baseline CD20 expression is assessed by FCM and reported as the percentage of CD20-positive blasts (among all blasts) in pretreatment BM or, more rarely, in PB. Based on research from trials conducted over the past decades, the ELN currently recommends at least 8 doses of rituximab for the management of CD20-positive B-ALL [15]. The GMALL 08/2013 therapy-optimization trial investigated risk-adapted, pediatric-inspired regimens in adults with ALL, regarding clinical risk factors and MRD-based stratification to guide stem cell transplantation (SCT) decisions. The trial incorporated up to 8 rituximab doses for all *BCR::ABL1*-negative B-ALL patients regardless of baseline CD20 expression [16]. In a subset of these patients, we studied baseline CD20 expression across compartments (BM and PB) and after prephase to monitor CD20 modulation, determine rituximab eligibility, and investigate the impact of CD20 expression level on early MRD response.

## Methods

### Patients

Patients (*n* = 274) aged 18–55 years with de novo B-ALL were treated within the pediatric-based, risk-adapted, MRD-stratified therapy-optimization trial of the GMALL 08/2013 in which rituximab was administered to all *BCR::ABL1*-negative B-ALL patients irrespective of baseline CD20 expression. The trial was registered with ClinicalTrials.gov: NCT02881086; EudraCT-Nr. 2013–003466-13. The analysis included 41% of patients with B-ALL/lymphoblastic lymphoma enrolled in GMALL 08/2013 for whom samples were available through the accompanying translational research program [17, 18]. *BCR::ABL1*-negative B-ALL patients aged 15–65 years (*n* = 174), enrolled in the previous GMALL 07/2003 trial before Amendment III (NCT00198991) and treated without rituximab, were used as a historical comparison cohort. All patients have provided written informed consent as part of their protocols. According to the Declaration of Helsinki, all research described herein was approved by the Frankfurt Research Ethics Board (188/15F). Further details on patients and analyses are provided in Table 1, Additional File 1: Materials and Methods, Additional File 2: Fig. S1.

**Table 1:**
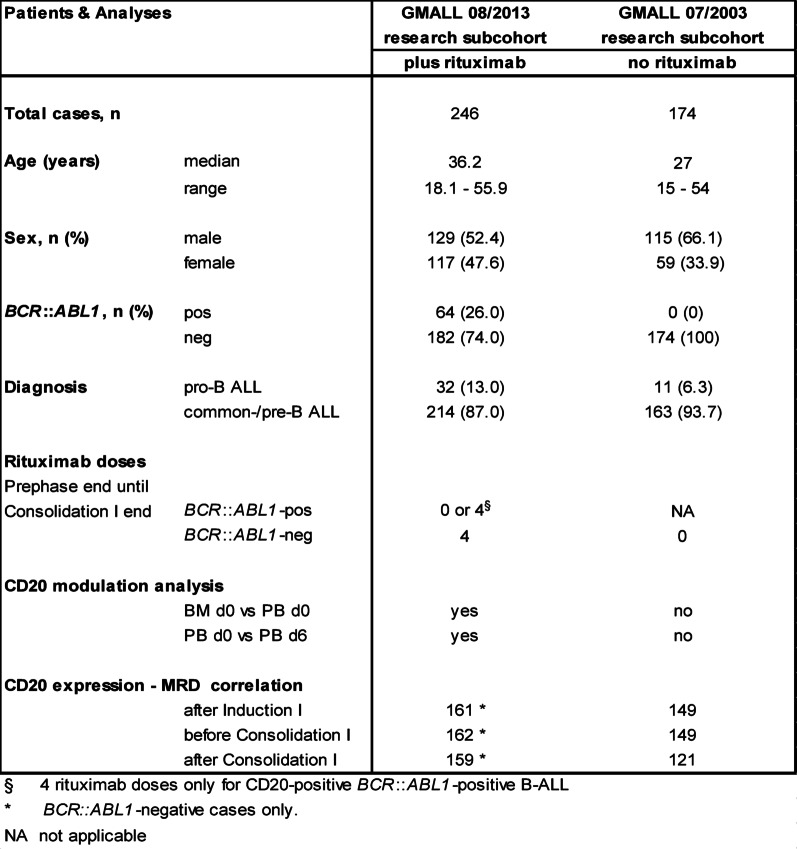
Patient characteristics and analysis information

### Early treatment and rituximab schedules within GMALL 08/2013

In the GMALL 08/2013 trial, all patients with newly diagnosed B-ALL, whether *BCR::ABL1*-negative or *BCR::ABL1*-positive, received an identical prephase regimen of cyclophosphamide plus dexamethasone. All *BCR::ABL1*-negative B-ALL subsequently received four doses of rituximab, irrespective of their baseline CD20 expression, in addition to their induction chemotherapy. The first dose was given on day 6, immediately after completion of prephase at the start of Induction I, followed by two additional doses during Induction I and a fourth dose during Consolidation I. Patients with *BCR::ABL1*-positive B-ALL received the four-dose rituximab regimen only if CD20-positive B-ALL was diagnosed (Additional File 3: Fig. S2). In the historical GMALL 07/2003 trial, all patients initially received an analogous prephase regimen; however, rituximab was not administered until the implementation of Amendment III. For our comparative analyses, only patients with *BCR::ABL1*-negative B-ALL enrolled before Amendment III were included (Additional File 2: Fig. S1).

### Multiparametric flow cytometry

Multiparametric FCM for immunophenotypic assessment of CD20 expression was performed in our reference laboratory following standardized instrument protocols established by the EuroFlow Consortium [19–23]. PB and BM were processed centrally using identical antibody panels, instrument settings, and gating strategies; paired samples obtained at the same time point were shipped and analyzed under identical conditions to minimize pre-analytical variability. Antibodies detailed in Additional File 4: Table S1 were used to quantify leukemic CD20 expression in GMALL 08/2013 cases using the monoclonal anti-CD20 antibody clone 2H7 (Biolegend, San Diego, CA, USA) and gating of the leukemic CD45^dim^/SSC^low^ cell fraction. Median CD20 fluorescence intensities (CD20 MFI) and percentages of CD20-positive B-ALL blasts (%CD20(+) blasts) were assessed. Immunophenotypes and percentages of the leukemic CD20 expression in historical GMALL 07/2003 cases were obtained from the trial sponsor as reported by the GMALL reference laboratory for flow cytometry (Additional File 1: Materials and Methods).

### Comparative CD20 modulation analysis

Because the prephase treatment was uniform, all cases, whether *BCR::ABL1*-negative or *BCR::ABL1*-positive, were eligible for comparative analyses of CD20 expression dynamics at baseline and the end of prephase. CD20 levels were quantified by FCM at baseline in BM samples at day 0 (BM d0) and/or PB samples at day 0 (PB d0) and post-prephase in peripheral blood at day 6 (PB d6) before the first rituximab administration (Table 1; Additional File 3: Fig. S2). Using the historical 20% threshold, B-ALL was defined as CD20-negative (< 20% of CD20(+) B-ALL blasts among total leukemic cells) or CD20-positive (≥ 20% of CD20(+) B-ALL blasts among total leukemic cells).

### MRD assessment and MRD-based risk stratification

All *BCR::ABL1*-negative B-ALL patients, whether CD20-positive or CD20-negative, uniformly received 4 rituximab doses from the start of Induction until Consolidation I in the GMALL 08/2013 trial. The treatment responses at stratification-relevant time points (after Induction I, before Consolidation I, and after Consolidation I) in GMALL 08/2013 patients (after one, three, and four doses of rituximab) and in GMALL 07/2003 patients (without rituximab treatment) were monitored through MRD analysis of leukemia-specific IG/TR gene rearrangements, obtained by real-time quantitative (RQ)-PCR in our laboratory [24–26]. IG/TR-based MRD values were used to categorize treatment responses as follows: molecular complete response (MolCR), defined as MRD negativity at a sensitivity of at least 1 × 10^− 4^; molecular intermediate response (MolIR), defined as MRD positivity that was either non-quantifiable or positive <1 × 10^− 4^; and molecular failure (MolFAIL), defined as MRD ≥1 × 10^− 4^ [27].

### Initial CD20 expression and correlation with early MRD response

In the GMALL 08/2013 trial, the respective IG/TR-based MRD values at stratification-relevant time points (after Induction I, before Consolidation I, and after Consolidation I) were correlated with either baseline CD20 expression in BM (as most commonly applied by the current standard approaches) or with the highest CD20 expression observed across BM and PB up to the end of prephase, to account for potential early CD20 upregulation. Cases of *BCR*::*ABL1*-positive B-ALL were excluded from MRD correlation analyses due to their distinct disease biology and divergent treatment schedules after the end of prephase. For comparison, a historical cohort of *BCR::ABL1*-negative B-ALL patients from the GMALL 07/2003 trial, who did not receive rituximab, was included. The percentage of CD20-positive blasts (%CD20(+) blasts) at baseline in BM was correlated with IG/TR-based MRD values after Induction I, before and after Consolidation I, which were highly comparable to the therapy schedules of the GMALL 08/2013 trial (Additional File 5: Fig. S3).

### Transcriptome analysis

Whole-transcriptome next-generation sequencing (RNA-seq) and subgroup allocation were performed as described [28, 29]. Molecular subtypes were predicted using the ALLCatchR classifier [30] (Additional File 1: Materials and Methods).

### Statistical analysis

The following statistical tests were applied: nonparametric two-tailed Mann-Whitney U test for unpaired-sample analysis of CD20 expression (BM d0 versus PB d0 and PB d0 versus PB d6); paired t*-test* for paired-sample analyses of CD20 expression (BM d0 versus PB d0 and PB d0 versus PB d6); Pearson’s chi-squared test with Monte Carlo simulation to compare early MRD response in B-ALL with < 20% and ≥ 20% CD20(+) blasts; nonparametric two-tailed Mann-Whitney U test and Kruskal-Wallis test to compare the distribution of early MRD responses with baseline CD20 expression in BCR::ABL1-like B-ALL. Logistic regression was used to evaluate associations with CD20 expression ≥ 20%. Univariable models were fitted for age, sex, white blood cell (WBC) count, and molecular subtype. The multivariable model included age per 10-year increase, sex, WBC per 10,000/µL, and molecular subtype. Odds ratios are reported with 95% confidence intervals. The global significance of molecular subtype in the multivariable model was assessed by likelihood-ratio testing against a reduced model without molecular subtype. GraphPad Prism 8.0.2 for Windows (GraphPad Software, San Diego, California, USA) and the R package for ChiSq, R version 4.4.0, 4.4.3 and 4.5.2 [31] were used. Statistical significance is indicated as: ns (not significant), *P* > 0.05, * (*P* ≤ 0.05), ** (*P* ≤ 0.01), *** (*P* ≤ 0.001), **** (*P* ≤ 0.0001).

## Results

### Cohort characteristics

We investigated CD20 expression modulation and its impact on MRD responses in 274 newly diagnosed adult B-ALL cases (Additional File 2: Fig. S1). Among these, 205/274 (74.8%) were *BCR::ABL1*-negative, comprising 164/205 (80.0%) common-/pre-B (c-/pre-B) ALL and 41/205 (20.0%) pro-B ALL cases, and 69/274 (25.2%) were *BCR::ABL1*-positive c-/pre-B ALL. *BCR::ABL1*-positive cases were included only in early CD20 modulation analyses (BM d0 vs. PB d0 and PB d0 vs. PB d6). Overall, in 1/173 (0.6%, d0) and 8/175 (4.5%, d6) PB samples no blasts were detected by FCM. A monocytoid lineage switch during prephase was defined according to Novakova et al. [32] as the presence of an intermediate B-monocytoid population characterized by a gradual decrease in CD19 expression accompanied by a gradual increase in at least one monocytic feature (CD14 expression, CD33 expression, or increased side scatter characteristics). Using this definition, a monocytoid lineage switch was observed in 23/205 (11.2%) *BCR::ABL1*-negative and 5/69 (7.2%) *BCR::ABL1*-positive B-ALL cases (overall 28/274, 10.2%) [32–34]. These cases were excluded from further analysis due to progressive loss of key lineage markers CD19 and CD20 (Additional File 2: Fig. S1). In total, 246 B-ALL cases, 182 *BCR::ABL1*-negative and 64 *BCR::ABL1*-positive, were included in the final analysis (Table 1).

### Global CD20 expression dynamics at baseline and during prephase

**Fig. 1:**
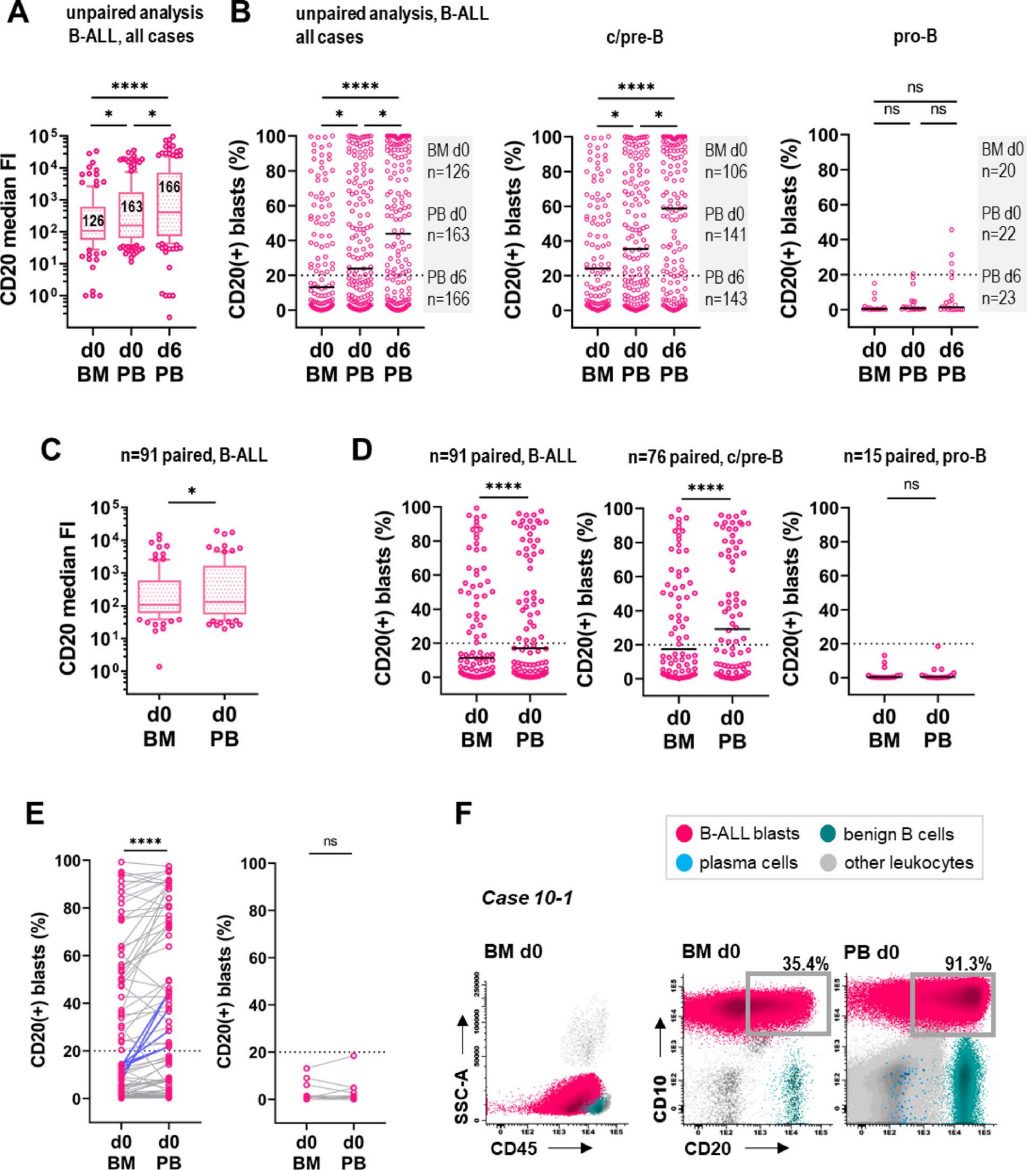
CD20 expression modulation in dependence on body compartment. **(A)** CD20 median fluorescence intensity (MFI) on blasts at baseline (d0) in bone marrow (BM) and peripheral blood (PB) and after prephase in PB (d6) in unpaired-sample analysis (*P* = 0.03; *P* = 0.0125; *P* < 0.0001, respectively); **(B)** Percentages of CD20(+) blasts in BM d0, PB d0 and PB d6 in total (*P* = 0.028; *P* = 0.023; *P* < 0.0001, respectively), in c-/pre-B ALL cases only (*P* = 0.046; *P* = 0.01; *P* < 0.0001, respectively) and in pro-B ALL only (ns, respectively) in unpaired-sample analysis. Case numbers are indicated on the plot´s right side; **(C)** CD20 MFI on blasts in 91 paired BM d0 and PB d0 samples (*P* = 0.0216); **(D)** Percentage of CD20(+) blasts in 91 paired samples of BM d0 and PB d0 in total (*P* < 0.0001), hereof in 76 c-/pre-B ALL cases (*P* < 0.0001), and 15 pro-B ALL cases (*P* = 0.8); **(E)** Rituximab-eligibility relevant CD20 upregulation: In 6/76 (7.9%) c-/pre-B ALL cases, the percentage of CD20(+) blasts in PB d0 exceeded the arbitrary 20% threshold, whereas the corresponding BM d0 samples were below this threshold (paired samples indicated by blue lines in the respective line plot); **(F)** Significant CD20 increase on B-ALL blasts between BM d0 and PB d0 [CD20 MFI at 312 versus 19198; percentage of CD20(+) blasts at 35.4% versus 91.3%] in a representative *BCR*::*ABL1*-positive B-ALL (case 10 − 1). The density plots visualize the distribution of detected events over staining intensity, with the highest density indicated by dark zones within the respective cell populations

At diagnosis, CD20 expression was significantly higher in PB than in BM (*P* = 0.03). During prephase treatment, CD20 expression increased further in PB (d0 vs. d6; *P* = 0.01), resulting in the highest CD20 MFI values at PB d6 (BM d0 vs. PB d6; *P* < 0.0001) (Fig. 1A). Similar findings were observed for the proportion of CD20-positive blasts (Fig. 1B). To address potential confounding bias and to test the robustness of these findings, paired BM and PB samples were analyzed (see below).

### Pretreatment CD20 expression modulation on B-ALL blasts upon transition from bone marrow to peripheral blood

Baseline CD20 expression was analyzed in 182 paired pretreatment BM and PB samples from 91 patients with *BCR::ABL1-*negative and *BCR::ABL1*-positive B-ALL whereas subsequent analyses of the correlation of CD20 expression and MRD response were restricted to *BCR::ABL1-*negative patients. CD20 MFI values were higher in PB d0 than BM d0 (*P* = 0.02) (Fig. 1C). Similarly, CD20(+) blast frequencies were significantly higher in PB d0 than in BM d0 overall (*P* < 0.0001) and specifically in c-/pre-B ALL (76 cases; *P* < 0.0001), but not in pro-B ALL (15 cases; *P* = 0.8) (Fig. 1D). Notably, 6/76 (7.9%) c-/pre-B ALL cases crossed the 20% threshold during transition from BM d0 to PB d0, meeting rituximab eligibility only in PB d0 (Fig. 1E). CD20 upregulation was surprisingly strong in some cases, exemplified by case 10 − 1 with 35.4% CD20(+) blasts in BM d0 vs. 91.3% in PB d0 (Fig. 1F).

### Prephase-induced upregulation of CD20 expression on B-ALL blasts

**Fig. 2:**
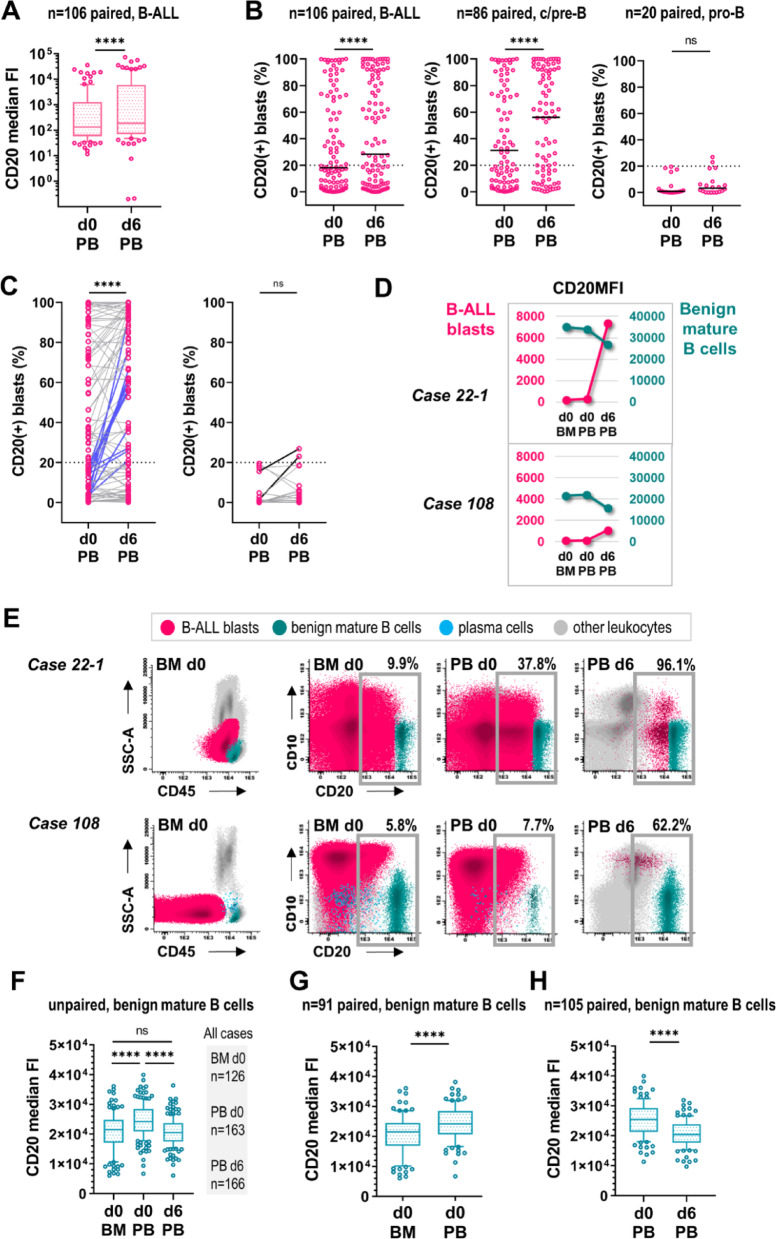
CD20 expression modulation under prephase treatment.** (A)** CD20 median fluorescence intensity (MFI) on blasts at baseline (d0) and after prephase (d6) in peripheral blood (PB) in paired-sample analysis of 106 B-ALL (*P* < 0.0001); **(B)** Percentages of CD20(+) blasts in PB d0 and PB d6 in paired-sample analysis of 106 B-ALL in total (*P* < 0.0001), hereof in 86 c-/pre-B ALL (*P* < 0.0001), and 20 pro-B ALL cases (*P* = 0.25); **(C)** Rituximab-eligibility relevant CD20 upregulation in 12/86 (13.9%) c-/pre-B ALL and 2/20 (10%) pro-B ALL with 20%-threshold-exceeding percentages of CD20(+) blasts in PB d6 (paired samples indicated by colored lines); **(D)** CD20 MFI values increase in blasts from BM d0 to PB d0 to PB d6, and in comparison, decrease in benign mature B cells from PB d0 to PB d6 (exemplified for cases 22 − 1 and 108); **(E)** Examples of CD20 modulation from BM d0 to PB d0 to PB d6 on blasts as measured by CD20 MFI and by percentages of CD20(+) blasts in case 22 − 1[CD20 MFI at 219 (BM d0), 301 (PB d0), and 7342 (PB d6); %CD20(+) blasts at 9.9% (BM d0), 37.8% (PB d0), and 96.1% (PB d6)], and in case 108 [CD20 MFI at 99.7 (BM d0), 112 (PB d0), and 1080 (PB d6); %CD20(+) blasts at 5.8% (BM d0), 7.7% (PB d0), and 62.2% (PB d6)]; **(F)** CD20 expression on benign mature B cells in unpaired-sample analysis: CD20 MFI significantly increase from BM d0 to PB d0 (*P* < 0.0001) and significantly decrease from PB d0 to PB d6 (*P* < 0.0001), returning to BM d0-levels (case numbers indicated on the plot´s right side). Significant increase of CD20 MFI on benign mature B cells in paired-sample analysis of **(G)** 91 B-ALL from BM d0 to PB d0 (*P* < 0.0001) and **(H)** 105 B-ALL from PB d0 to PB d6 (*P* < 0.0001)

Early therapy CD20 modulation was further analyzed in 212 paired PB d0 (baseline) and PB d6 (post-prephase) samples from 106 patients with *BCR::ABL1-*negative and *BCR::ABL1*-positive B-ALL. CD20 MFI values and CD20(+) blast percentages increased significantly during prephase (both *P* < 0.0001) (Fig. 2A-B). Briefly, the percentages of CD20(+) blasts were significantly higher in PB d6 than in PB d0 in c-/pre-B ALL (86 cases; *P* < 0.0001) but not in pro-B ALL (20 cases; *P* = 0.25) (Fig. 2B). Notably, the CD20(+) blast frequencies exceeded the 20% threshold in 2/20 (10.0%) pro-B and 12/86 (13.9%) c-/pre-B ALL cases from PB d0 to PB d6 (Fig. 2C). In summary, overall CD20 upregulation during early therapy was observed across compartments and over time as illustrated by two representative c-/pre-B ALL cases (Fig. 2D-E). In contrast, benign mature B cells showed divergent CD20 modulation dynamics. In unpaired analyses, CD20 MFI was higher in PB d0 (*n* = 163) than BM d0 (*n* = 126) (*P* < 0.0001) but decreased at PB d6 compared to PB d0 (*P* < 0.0001), returning to BM-like levels (Fig. 2F). Paired analyses confirmed CD20 upregulation from BM d0 to PB d0 (*n* = 91; *P* < 0.0001) and downregulation from PB d0 to PB d6 (*n* = 106; *P* < 0.0001) (Fig. 2G-H).

### Clinical and molecular factors associated with CD20 expression and modulation

Baseline CD20 expression differed substantially between molecular subgroups (Additional File 6: Fig. S4A). In addition to KMT2A-rearranged B-ALL, cases from CDX2/UBTF, ZNF384- and TCF3::PBX1 rearranged subtypes showed consistently low CD20 expression, whereas BCR::ABL1-positive, BCR::ABL1-like, low-hypodiploid, hyperdiploid, and PAX5alt B-ALL were characterised by high CD20 expression. Similar differences were observed for CD20 modulation during prephase treatment, with little or no CD20 upregulation in molecular subgroups characterised by low baseline expression. Because several subgroups contained only small numbers of cases, these analyses were considered exploratory and descriptive rather than suitable for formal subgroup-specific statistical testing.

To understand the contribution of molecular subgroups and clinical disease characteristics, we performed logistic regression analyses using CD20 status as the binary outcome, defined as the highest available CD20 expression value ≥ and < 20% according to the predefined cutoff. In univariable logistic analyses, both sex and WBC count were associated with CD20 expression levels ≥ 20%. However, neither variable remained significant after adjustment for molecular subgroup in multivariable regression models, suggesting that these associations largely reflected differences in molecular subtype composition (Additional File 6: Fig. S4B-C, Additional File 7: Table S2). Molecular subtype was significantly associated with CD20 status overall. This association was mainly driven by the strong enrichment of CD20-negative cases among KMT2A-rearranged leukemia. Other subtype-specific comparisons did not reach statistical significance, likely reflecting small subgroup sizes and wide confidence intervals. Consistent results were obtained in linear regression analyses using continuous CD20 expression as the outcome, supporting molecular subgroup irrespective of age, sex and WBC count as the strongest independent correlate of CD20 expression (not shown).

### CD20 expression levels and early MRD response under rituximab in GMALL 08/2013 patients

We evaluated the impact of baseline CD20 expression on rituximab efficacy by analyzing IG/TR-based early-phase MRD responses in *BCR::ABL1*-negative B-ALL. Among 182 patients, 76 (41.8%) had < 20% of CD20(+) blasts (range 0.0–19.9%, median 3.8%) and 106 (58.2%) had ≥ 20% of CD20(+) blasts (range 21.2–100.0%, median 78.6%) (Additional File 8: Fig. S5 A-B). All patients uniformly received four rituximab doses: one at the start of Induction I, two additional doses before, and one more at Consolidation I (Additional File 3: Fig. S2; Additional File 5: Fig. S3).

**Fig. 3:**
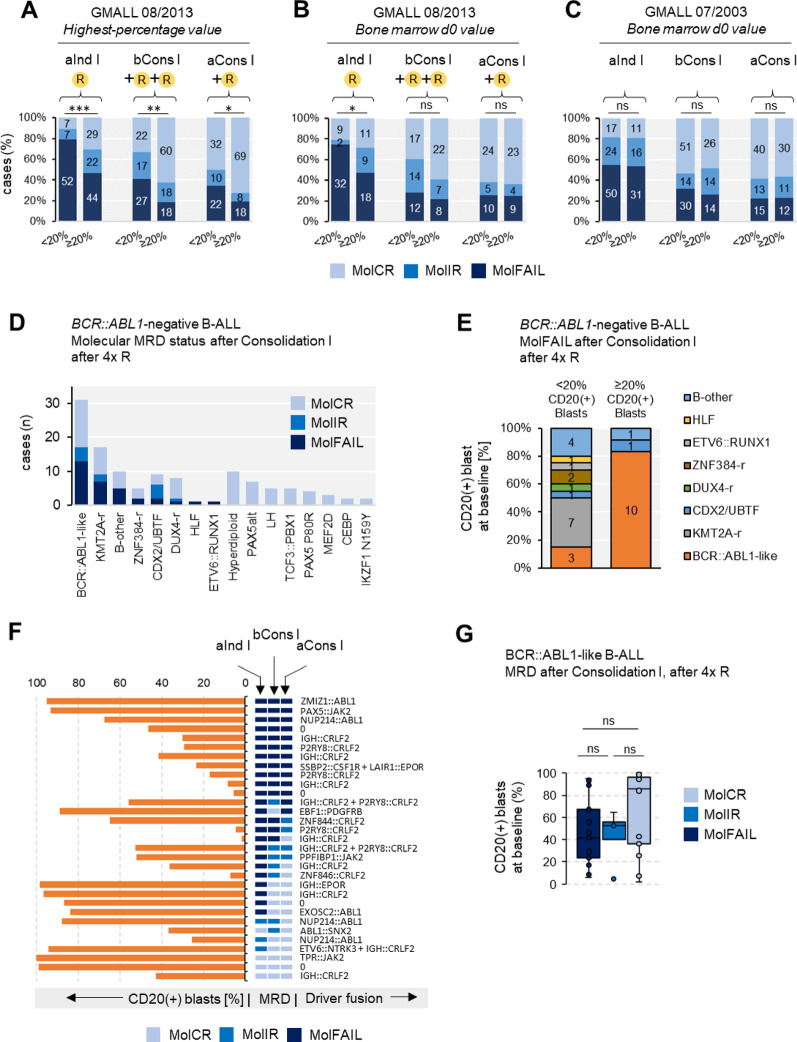
MRD responses.** (A)** In the GMALL 08/2013 trial cohort treated with rituximab, MRD responses were analyzed using the highest measured percentage of CD20-positive blasts. There were significant differences in MRD response between patients with highest measured CD20 < 20% and those with highest measured CD20 ≥ 20%, after Induction I, before and after Consolidation I; **(B)** When MRD dynamics were evaluated using bone marrow day-0 (BM d0) CD20 values, significant differences between the < 20% and ≥ 20% CD20 groups were observed only after Induction I, not at later time points; **(C)** In contrast, in the GMALL 07/2003 trial cohort without rituximab treatment, MRD responses did not differ significantly between patients with baseline CD20 < 20% vs. ≥20%, suggesting that the correlation seen in **(A–B)** is rituximab-dependent; **(D)** Among *BCR::ABL1*-negative B-ALL patients who remained MRD-positive (MolFAIL) after Consolidation I, the most common molecular subtypes were BCR::ABL1-like, KMT2A-rearranged, and B-other; **(E)** and BCR::ABL1-like cases predominated among the CD20-positive MolFAIL cases; **(F)** Overall, among the 31 BCR::ABL1-like cases, 14 (45.0%) were in MolCR, 4 (13.0%) in MolIR, and 13 (42.0%) in MolFAIL after Consolidation I; **(G)** and their baseline CD20 expression was variable but the levels did not differ significantly between molecular MRD response groups (Mann-Whitney test: MolCR vs. MolIR, *P* = 0.4; MolCR vs. MolFAIL, *P* = 0.2; MolIR vs. MolFAIL, *P* = 0.9; Kruskal-Wallis test: *P* = 0.36)

As previously demonstrated, baseline CD20 expression can vary significantly depending on body compartment and is influenced by prephase-drug exposure (Figs. 1 and 2). Using each patient´s highest CD20(+) percentage from BM d0, PB d0 or PB d6, patients with ≥ 20% CD20(+) blasts showed significantly more favorable MRD response categories, including higher rates of MolCR and lower rates of MolFAIL after Induction I, before Consolidation I, and after Consolidation I compared with those with < 20% CD20(+) blasts (*P* = 0.0005, *P* = 0.0012, *P* = 0.017, respectively). CD20 expression and molecular MRD data were available for 161 patients after Induction I, 162 before Consolidation I, and 159 after Consolidation I. (Fig. 3A).

The most pronounced difference was observed after Induction I: 78.8% of patients with < 20% of CD20(+) blasts exhibited MolFAIL, compared with 46.3% of those with ≥ 20%. By the end of Consolidation I, MolCR was reached in 50.0% of patients with < 20% CD20(+) blasts compared with 72.6% of those with ≥ 20% CD20(+) blasts.

Although the number of evaluable patients was lower because PB d6 material was not available for all cases and 4.6% of samples had no detectable PB blasts at this time point, comparable associations were observed when the analysis was restricted to PB d6 samples (*P* = 0.0019, *P* = 0.0005, *P* = 0.0005, respectively).

Analysis restricted to BM d0 alone within the GMALL 08/2013 cohort, reflecting routine-diagnostics procedures and enabling comparison with GMALL 07/2003 B-ALL patients not treated with rituximab (see below), revealed significant differences only after Induction I (*P* = 0.014), but not before or after Consolidation I (Fig. 3B). Here, 74% of patients with < 20% CD20(+) blasts exhibited MolFAIL after Induction I versus 47% with ≥ 20%. MolCR after Consolidation I was similar between groups with ≥ 20% or < 20% CD20(+) blasts (64% vs. 61%). BM d0 CD20 expression and molecular MRD data were available for 81 patients after Induction I, 80 before Consolidation I, and 75 after Consolidation I. These results indicate that MRD response to rituximab correlates more strongly with the highest observed CD20 level across compartments and time points than with baseline BM expression.

### Baseline CD20 expression levels and early MRD response in a rituximab-free historical GMALL 07/2003 cohort

MRD responses were analysed after Induction I (*n* = 149), before Consolidation I (*n* = 149), and after Consolidation I (*n* = 121) in *BCR::ABL1*-negative B-ALL patients from the historical GMALL 07/2003 trial, who did not receive rituximab and had only a single baseline BM CD20 measurement given as percentage of CD20(+) blasts. MRD responses did not differ between patients with < 20% and ≥ 20% CD20(+) blasts at any time point (*P* = 1.0, *P* = 0.2, *P* = 0.97, respectively) (Fig. 3C).

### MRD persistence under rituximab despite high CD20 expression: enrichment of BCR::ABL1-like B-ALL

RNA-seq data were available for 184/246 (75.0%) cases, of which 49 (26.6%) were *BCR::ABL1*-positive by RT-PCR (and had the BCR::ABL1 molecular signature) and 135 (73.4%) *BCR::ABL1*-negative cases with other molecular signatures (Additional File 8: Fig. S5C). These *BCR::ABL1*-negative cases showed the following molecular-subtype distribution based on RNA-seq data (Additional File 8: Fig. S5D): 32 (23.7%) BCR::ABL1-like, 21 (15.6%) KMT2A-r, 12 (8.9%) Hyperdiploid, 10 (7.4%) CDX2/UBTF, 9 (6.7%) DUX4-r, 8 (5.9%) PAX5alt, 6 (4.4%) TCF3::PBX1, 5 (3.7%) LH, 5 (3.7%) ZNF384-r, 4 (3.0%) PAX5 P80R, 3 (2.2%) CEBP, 3 (2.2%) MEF2D, 3 (2.2%) IKZF1 N159Y, 1 (0.7%) ETV6::RUNX1, 1 (0.7%) HLF, and 12 (8.9%) B-other. IG/TR-based MRD data after Consolidation I were available for 120 of these *BCR::ABL1*-negative patients. Of these, 77 (64.2%) achieved MolCR, 11 (9.2%) MolIR, and 32 (26.7%) remained in MolFAIL. Most MolFAIL cases (25/32, 78.0%) were concentrated in BCR::ABL1-like (13/32, 40.6%), KMT2A-r (7/32, 21.9%), and B-other (5/32, 16.5%) subtypes (Fig. 3D). Among these MolFAIL cases 20/32 (62.5%) were CD20-negative (Fig. 3E) and classified as KMT2A-r (7/20, 35.0%), B-other (4/20, 20.0%), BCR::ABL1-like (3/20, 15.0%), ZNF384-r (2/20, 10.0%), and CDX2/UBTF, DUX4-r, ETV6::RUNX1, and HLF (each 1/20, 5%). Among the remaining 12/32 (37.5%) CD20-positive MolFAIL cases, 10/12 (83%) were BCR::ABL1-like, 1/12 CDX2/UBTF, and 1/12 B-other (8.3%, respectively) (Table 2).

**Table 2:**
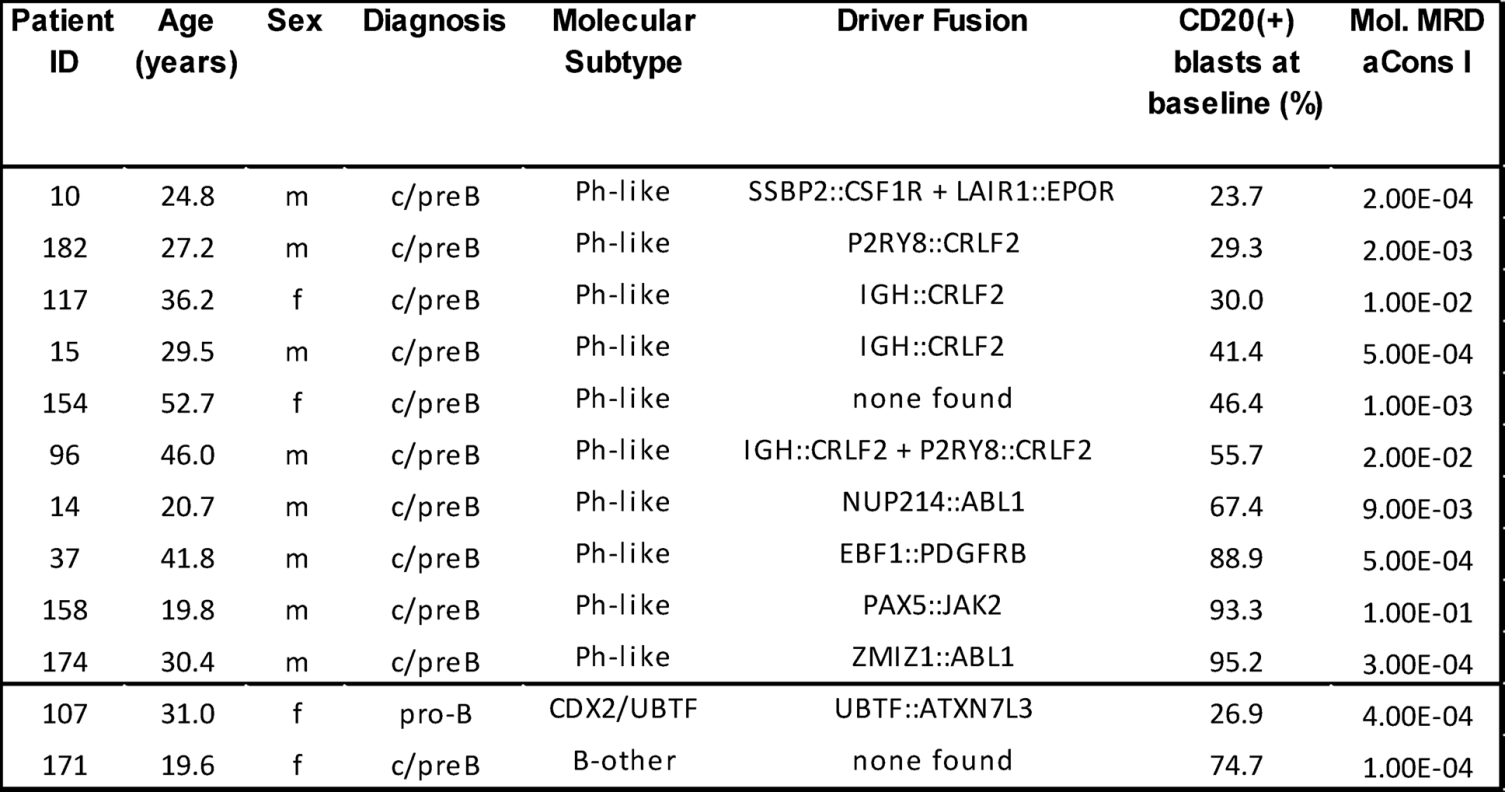
Molecular non-responder (MolFAIL) after Consolidation I among the CD20-positive *BCR::ABL1*-negative B-ALL

Given the high prevalence of BCR::ABL1-like cases among MRD non-responders, we examined MRD dynamics of all 31 BCR::ABL1-like patients (Fig. 3F). After Consolidation I, 14 (45.0%) were in MolCR, 4 (13.0%) in MolIR, and 13 (42.0%) in MolFAIL. Baseline CD20 expression varied: 6 patients were CD20-negative (range 1.7%−17.1%, median 6.4% of CD20(+) blasts) and 25 patients were CD20-positive (range 23.7–100.0%, median 64.8% of CD20(+) blasts). CD20 expression levels did not differ significantly across MRD groups (Mann-Whitney test: MolCR vs. MolIR, *P* = 0.4; MolCR vs. MolFAIL, *P* = 0.2; MolIR vs. MolFAIL, *P* = 0.9; Kruskal-Wallis test: *P* = 0.36), though MolCR patients demonstrated a trend towards higher baseline CD20 expression (Fig. 3G).

Among CD20-positive BCR::ABL1-like MolFAIL patients after Consolidation I (10/12, 83%) who received four upfront rituximab doses, only 4 achieved MolCR after blinatumomab, 6 patients relapsed, requiring tertiary immunotherapy, and 4 suffered subsequent second relapses. The 4 patients achieving MolCR after blinatumomab proceeded to allogeneic stem cell transplantation and remained MRD-negative during follow-up (22–103 months; median 53.5 months).

## Discussion

The addition of rituximab to standard chemotherapy has improved early treatment responses and remission duration in CD20-positive B-ALL [4, 6, 8, 9, 35–37]. Rituximab eligibility is conventionally based on the percentage of CD20-positive blasts in diagnostic BM, with the ≥ 20% cut-off used as an arbitrary threshold [9]; however, CD20 expression can change dynamically on blasts following body-compartment transitions and corticosteroid exposure. Prior pediatric studies demonstrated corticosteroid-induced CD20 upregulation during early therapy, attributed to prednisolone-mediated maturation [12, 13]. Additionally, a recent study on pediatric B-ALL observed an increase in CD20 MFI between diagnosis and day 15, showing that both CD20 values were associated with RFS and OS, using cut-offs of > 8.08 at diagnosis and > 28.65 at day 15. In this study, CD20 expression appeared to be a poor prognostic feature in pediatric B-ALL [38]. CD20 upregulation following dexamethasone-containing prephase was also observed in six of eight immunophenotyped adult B-ALL patients in the UKALL14 trial [11]. Our findings align with these data and add new perspectives to this topic. In our larger adult cohort, we identified two stages of leukemic CD20 upregulation relevant for rituximab eligibility: lower CD20 expression in BM versus PB at baseline, and significant CD20 increases in PB during cyclophosphamide- and dexamethasone-containing prephase. Together, these data provide strong evidence that early-therapy CD20 upregulation is a common phenomenon in adult B-ALL and that CD20 modulation is influenced by microenvironmental factors (BM vs. PB) and corticosteroid exposure. We also observed a monocytoid lineage switch during prephase in approximately 10% of cases, a frequency comparable to that reported in pediatric B-ALL [32–34].

Furthermore, in our exploratory analysis, CD20 expression appeared to vary across molecular subgroups, suggesting a potential subgroup-specific regulation of CD20 expression. Larger cohorts are needed to systematically validate these findings and determine their clinical relevance.

MRD is an accurate surrogate parameter for assessing treatment efficacy in ALL [27, 39–41]. In our study, CD20-negative B-ALL, defined by 20% threshold, showed slow MRD response, suggesting limited benefit from CD20-targeting therapy, whereas higher baseline CD20 expression significantly correlated with improved early MRD response in rituximab-treated patients. No such association was found in the historical cohort between CD20-negative and CD20-positive cases that did not receive rituximab.

The prognostic significance of CD20 expression in adult B-ALL remains controversial. While some studies reported an adverse impact of CD20 expression in the pre-rituximab era, others failed to confirm an association with MRD response or clinical outcome [4, 5, 42]. Our findings in the historical GMALL 07/2003 cohort are consistent with the latter observations and suggest that CD20 expression may be more relevant as a predictive biomarker for CD20-directed therapy than as an independent prognostic marker. This interpretation is supported by the association between CD20 expression and MRD response observed in rituximab-treated patients, which was absent in the historical cohort treated without rituximab.

In our study, the 20% threshold of CD20-expressing blasts has been surpassed from diagnostic BM to PB (7.9% of c-/pre-B ALL) and from diagnostic PB to post-prephase PB (10.0% of pro-B ALL and 13.9% of c-/pre-B ALL). These findings challenge reliance on diagnostic bone marrow alone for rituximab eligibility and suggest reassessment of CD20 expression in PB at the end of prephase. This strategy would identify patients with prephase-driven CD20 upregulation above the 20% threshold who may benefit from rituximab. A rationale for this adapted strategy, as well as the potential impact on outcomes, needs further investigation in prospective trials.

Importantly, using the highest measured percentage of CD20(+) blasts across compartments and time points or blood on day 6 instead of the baseline BM value provides superior prediction of early MRD response and more accurately reflects the impact of leukemic CD20 expression levels on MRD dynamics during rituximab treatment.

From a clinical perspective, these findings support viewing CD20 expression as a dynamic biomarker. Assessment immediately before rituximab administration may provide a more relevant estimate of target availability than assessment at diagnosis alone. Such an approach could identify patients who acquire clinically relevant CD20 expression during prephase treatment and may therefore benefit from anti-CD20 therapy despite low baseline expression. Conversely, patients with persistently low or absent CD20 expression, particularly within molecular subgroups characterised by intrinsically low CD20 levels and little CD20 upregulation, may derive less benefit from rituximab. Although this hypothesis requires prospective validation, our data support the concept of a more individualised, biomarker-guided use of anti-CD20 therapy in adult B-ALL.

Delayed MRD responses under rituximab are expected in *KMT2A*-rearranged B-ALL due to low or absent CD20 expression, whereas B-ALL patients with high baseline CD20 expression are theoretically more likely to respond well to anti-CD20 therapy. Thus, the disproportionate enrichment of BCR::ABL1-like cases with moderate to high baseline CD20 expression among molecular non-responders after Consolidation I was unexpected. These patients accounted for 41% of *BCR::ABL1*-negative non-responders, although their overall prevalence in our study sub-cohort of *BCR*::*ABL1*-negative cases was considerably lower (23.7%). This suggests that a subset of CD20-positive BCR::ABL1-like B-ALL may harbor intrinsic adverse features that diminish the expected benefit of rituximab. These cases may warrant closer clinical attention and additional investigation to identify underlying resistance mechanisms and guide more tailored therapeutic approaches.

Despite great advances in CD19- and CD22-directed therapies, anti-CD20 approaches remain clinically highly relevant. Recently, inotuzumab ozogamicin showed increased toxicity in frontline B-ALL therapy in the Alliance A041501 trial, likely related to its immunosuppressive effects [43]; thus, targeted therapeutic alternatives remain warranted. Beyond rituximab, multiple anti-CD20 agents, including monoclonal antibodies, bispecific antibodies, and antibody–drug conjugates, are in various stages of clinical approval. Ofatumumab, an anti-CD20 monoclonal antibody, is part of the evolving landscape of therapies for patients with B-ALL [44]. Notably, several CD20×CD3 bispecific antibodies (e.g., mosunetuzumab, glofitamab, epcoritamab, odronextamab) have shown substantial activity in mature B-cell malignancies [45]. Glofitamab is used for treating diffuse large B-cell lymphoma and has been successfully tested in combination with polatuzumab vedotin in three patients with refractory Burkitt lymphoma [46]. Beyond CD3-engaging BsAbs, other bispecific formats under investigation include CD20xCD19, CD20xCD47, and CD20xCD28 antibodies [47]. All these agents have the potential and may be extended to the treatment of CD20-positive B-ALL.

## Conclusions

We correlated CD20 expression with early treatment response in adult B-ALL of the GMALL 08/2013 trial, in which all *BCR::ABL1*-negative patients received rituximab irrespective of baseline CD20 levels, and CD20 was assessed in a standardised manner. We demonstrate that CD20 expression is dynamically regulated during the early disease course, increasing as blasts transit from bone marrow to peripheral blood and further rising after a 5-day cyclophosphamide- and dexamethasone-containing prephase. In our study, the highest assessed CD20 expression level, rather than the baseline bone marrow value, best predicts MRD response to rituximab, and a subset of initially CD20-negative patients may become rituximab-eligible during early treatment phases. These findings support longitudinal reassessment of CD20 (e.g., after prephase, including peripheral blood) and suggest a potentially practice-changing approach to anti-CD20 therapy in B-ALL.

However, a subset of CD20-positive *BCR::ABL1*-like B-ALL remains refractory despite upfront rituximab, indicating that CD20 expression alone does not always overcome adverse biology and that these patients require additional therapeutic consideration.

## Supplementary Information

Supplementary Material 1

## Data Availability

Flow cytometry raw data can be requested by contacting the corresponding author and viewed following the current national and international data safety regulations. Primary RNA high-throughput sequencing data have been deposited in the European Genome Phenome Archive (EGAS00001006107).

## Abbreviations

B-ALL: B-cell acute lymphoblastic leukemia
BsAbs: Bispecific antibodies
d0: Day zero, baseline
d6: Day six, after prephase
EFS: Event-free survival
FCM: Multiparametric flow cytometry
IG/TR: Immunoglobulin/T-cell gene rearrangements
mAb: Monoclonal antibody
MolCR: Molecular complete remission
MolFAIL: Molecular failure
MolIR: Molecular intermediate response
MRD: Measurable residual disease
OS: Overall survival
PB: Peripheral blood
