# Supplementary material for: CD20 expression dynamics in adult B-cell acute lymphoblastic leukemia and impact on anti-CD20 treatment
Source: J Hematol Oncol. 2026 Jul 20;19:52. doi: 10.1186/s13045-026-01832-4 (PMC13386618; doi:10.1186/s13045-026-01832-4)
Supplement: Supplementary file 1 — Supplementary Material 1 [file 13045_2026_1832_MOESM1_ESM.docx]

**Additional File 1**

**Materials and Methods**

**CD20 Expression Dynamics in Adult B-Cell Acute Lymphoblastic Leukemia and Impact on Anti-CD20 Treatment**

Monika Szczepanowski^1,13^, Johanna Richter^1,11^, Sonja Bendig^1,12,13^, Lorenz Bastian^1,12,13^, Miriam Kelm^1,13^, Veronika Beck^1^, Thomas Beder^1,12,13^, Guranda Chitadze^1,12,13^, Michaela Kotrová^1,13^, Johannes Duell^2^, Christoph Faul^3^, Johannes Gärtner^4^, Alina Hartmann^1,12,13^, Britta Kehden^1,13^, Eva Maria Murga Penas^5^, Martin Neumann^1,13^, Matthias Ritgen^1,13^, Wiebke Schrader^1,13^, Björn Steffen^6^, Heiko Trautmann^1,13^, Andreas Viardot^7^, Thomas Burmeister^8^, Claudia D. Baldus^1,12,13^, Stefan Schwartz^9,10^*, Nicola Gökbuget^6^*, Monika Brüggemann^1,12,13^*

**Table of Contents**

[1 Patients 2](#_Toc225761651)

[2 Details on analyses regarding differences in therapy elements 2](#_Toc225761652)

[3 Routine laboratory procedures 3](#_Toc225761653)

[4 Measurable residual disease assessment 3](#_Toc225761654)

[**4.1** **MRD markers** 3](#_Toc225761655)

[**4.2** **MRD-based stratification** 4](#_Toc225761656)

[5 Flow cytometry 4](#_Toc225761657)

[**5.1** **Routine Multiparametric Flow Cytometry** 4](#_Toc225761658)

[**5.2** **In-house Reference Multiparametric Flow Cytometry** 4](#_Toc225761659)

[**5.2.1** **Instruments** 4](#_Toc225761660)

[**5.2.2** **Software** 5](#_Toc225761661)

[**5.2.3** **Sample processing** 5](#_Toc225761662)

[**5.2.4** **Antibody panels** 5](#_Toc225761663)

[6 Transcriptome Analysis 6](#_Toc225761664)

[7 References 6](#_Toc225761665)

# **1 Patients**

Adult patients (n=274) aged 18-55 years and newly diagnosed with B cell precursor lymphoblastic leukemia (B-ALL) were prospectively treated according to therapy protocols of the German Multicentric Acute Lymphoblastic Leukemia (GMALL) 08/2013 trial (EUDRACT2013-003466-13, govNCT02881086) with rituximab administered to all *BCR*::*ABL1*-negative B-ALL both with ≥20% and <20% CD20(+) blasts at diagnosis as well as to *BCR::ABL1*-positive B-ALL with ≥20% CD20(+) blasts. Patients were stratified into risk groups as defined by (i) the Stratification I criteria, including initial leukocyte count, immunological subtype, *BCR*::*ABL1*, and *KMT2A* status, and (ii) the Stratification II criteria, including MRD response before and after Consolidation I. A historical cohort of 174 patients aged 15-65, diagnosed with *BCR*::*ABL1*-negative B-ALL, who were enrolled in the GMALL 07/2003 trial (govNCT00198991) before Amendment III and did not receive rituximab, was used for comparison. Importantly, patients aged 55-65 at diagnosis were only enrolled in GMALL 07/2003 if they were biologically younger. All patients have provided written informed consent as part of their protocols. According to the Declaration of Helsinki, all research described herein was approved by the Frankfurt Research Ethics Board (188/15F). Table 1 and Additional File2: Fig. S1 provide an overview of patients and analyses.

# **2 Details on analyses regarding differences in therapy elements**

In the context of our CD20 expression modulation studies within the GMALL 08/2013 trial, it is important to note that all B-ALL cases, both *BCR::ABL1*-negative and *BCR::ABL1*-positive, standard risk (SR) and high risk (HR) at Stratification I, were uniformly treated and remained rituximab-naïve until the end of prephase (day 6). Thus, all results regarding comparisons of CD20 expression at baseline (day 0, d0) and after prephase (day 6, d6) in (i) unpaired samples of bone marrow (BM) d0, peripheral blood (PB) d0 and PB d6, and (ii) paired samples of BM d0 versus PB d0 and PB d0 versus d6, rely on identically treated cohorts (Table 1, Additional File 3: Fig. S2).

The first differences in rituximab administration and other therapy elements between *BCR*::*ABL1*-positive and *BCR::ABL1*-negative patients occur at the beginning of Induction I. Thus, all patients with *BCR*::*ABL1*-positive B-ALL were excluded from further analyses involving later time points, e.g., from correlations of the MRD response under rituximab with baseline CD20 expression levels at early therapy phases (after Induction I, before and after Consolidation I).

On the other side, all patients with *BCR*::*ABL1*-negative B-ALL both with initially <20% CD20(+) and ≥20% CD20(+) blasts were uniformly treated until the second stratification time point (after Consolidation I) receiving 4 doses of rituximab, which allowed for comparative analyses of MRD response within this patient group at early therapy phases (after Induction I, before and after Consolidation I).

# **3 Routine laboratory procedures**

Mononuclear cells from initial and follow-up bone marrow and/or peripheral blood samples were enriched by density gradient centrifugation and subjected to DNA extraction.

DNA for clone-specific immunoglobulin/T-cell receptor (IG/TR) gene rearrangement screening was extracted from diagnostic samples, and for measurable residual disease (MRD) assessment via clone-specific IG/TR gene rearrangement real-time quantitative PCR (RQ-PCR) from follow-up samples following standard procedures (AllPrep, Qiagen, Hilden, Germany).

RNA for transcriptome analysis via high-throughput next-generation RNA sequencing (RNA-seq) was extracted from mononuclear cells following standard procedures (Trizol, Life Technologies, Carlsbad, CA).

The standard molecular assessment for *BCR::ABL1*, *KMT2A::AFF1*, and *KMT2A::ENL* gene fusions was achieved by performing breakpoint-specific quantitative reverse transcription (qRT)-PCRs in the central reference laboratory of the GMALL study group at the Department of Hematology/Oncology, Charité - Universitätsmedizin Berlin, Campus Benjamin Franklin, Berlin, Germany (T. Burmeister lab).

# **4 Measurable residual disease assessment**

## **4.1 MRD markers**

The leukemia burden (MRD) was monitored at defined therapy time points by leukemia clone-specific RQ-PCR. Clone-specific molecular markers for RQ-PCR were routinely established for all patients at diagnosis based on the unique IG/TR gene rearrangements (1–7).

## **4.2 MRD-based stratification**

MRD values at early therapy time points after Induction I (aInd I), before (bCons I), and after Consolidation I (aCons I) were used to stratify patients as molecular complete response (MolCR, defined as MRD negativity at assay sensitivity of at least 1x10^-4^), molecular intermediate response (MolIR, MRD positive non-quantifiable or MRD positive <1x10^-4^), and molecular failure (MolFAIL, MRD ≥1x10^-4^) (8).

# **5 Flow cytometry**

## **5.1 Routine Multiparametric Flow Cytometry**

Diagnostic standard immunophenotyping for GMALL 07/2003 and GMALL 08/2013 was performed by flow cytometry in the central reference laboratory of the GMALL study group at the Department of Hematology, Oncology and Cancer Immunology, Campus Benjamin Franklin, Charité - Universitätsmedizin, Berlin, Germany (S. Schwartz lab). Briefly, the leukemic cell fraction was always enriched by density-gradient centrifugation (Biocoll, Biochrom GmbH, Berlin, Germany) before further processing. Routine flow cytometry (Navios, Beckmann Coulter, Krefeld, Germany) was performed as previously described with some modifications (i.e., 5-color staining, CD45/SSC gating strategy) (9). Routine determination of the percentage of CD20(+) B-ALL blasts within the GMALL 07/2003 cohort was based on the use of the monoclonal antibody clone L27 (BD Bioscience, San Jose, California, USA) and gating of the leukemic, CD45^dim^/SSC^low^ cell fraction.

## **5.2 In-house Reference Multiparametric Flow Cytometry**

A second set of multiparametric flow cytometry measurements of diagnostic samples (BM d0 and PB d0) and PB d6 was performed in the central reference laboratory for the German NHL- and ALL-BFM Study Groups of pediatric ALL and NHL at the 2nd Department of Internal Medicine, University Hospital Schleswig-Holstein, Campus Kiel, Kiel, Germany (M. Brüggemann lab).

### **5.2.1 Instruments**

Cell acquisition was performed on the BD FACSCanto II cytometer and in the second project period on the BD FACSLyric cytometer (BD, Heidelberg, Germany). Both instruments were equipped with the 405 nm violet laser, 488 nm blue laser, and 640 nm red laser, respectively.

### **5.2.2 Software**

The operating software was BD FACSDIVA on the BD FACS Canto II instrument and BD FACSSuite on the BD FACSLyric instrument (BD Biosciences, San Jose, CA, USA). Detailed analyses of CD20 and other surface antigen expression were conducted using the Infinicyt software (Cytognos, S.L., Salamanca, Spain; since 2022 part of BD Life Sciences, San Jose, CA, USA).

### **5.2.3 Sample processing**

Nonmanipulated heparinized or EDTA-anticoagulated peripheral blood and bone marrow aspirate samples were used for surface CD20 expression analysis by multiparametric flow cytometry. Antibody staining procedures and flow cytometer instrument settings were applied according to standardized operating procedures published by the EuroFlow Consortium (10–13). Briefly, samples were washed twice in phosphate-buffered saline (PBS)/0.2% BSA/0.09% NaN_3_, stained according to the extracytoplasmic staining protocol, and subjected to red blood cell lysis using the BD FACS^TM^ Lysing Solution (BD Biosciences, Heidelberg, Germany). All antibodies, conjugated fluorochromes, antibody clones, and the respective manufacturers are listed in Table S1. All samples were measured at the medium sample flow rate (60 µL/min).

### **5.2.4 Antibody panels**

The antibodies and antibody panels used for the indicated research are listed in Additional File 4: Table S1 and described below.

**CD20 Expression Antibody Panel 1.** The 3-tube 8-color antibody panel was designed based on recommendations from the Associazione Italiana Ematologia Oncologia Pediatrica (AIEOP) / International Berlin-Frankfurt-Münster (iBFM) ALL Trial Group and the EuroFlow Consortium (14, 15). Flow cytometry measurements were conducted on the BD FACSCanto II instrument (BD, Heidelberg, Germany).

**CD20 Expression Antibody Panel EuroFlow (EF).** The 3-tube 8-color antibody panel was entirely adopted from the EuroFlow Consortium protocol (15) in the second period of the research project. The flow cytometry measurements were conducted on the BD FACSLyric instrument.

# **6 Transcriptome Analysis**

RNA high-throughput sequencing and subgroup allocation were performed as described (16–18). Briefly, RNA samples with a RIN < 6 were excluded from further processing. Libraries were prepped using the TruSeq RNA Library Prep Kit (Illumina) and sequenced at 2x100 bp paired-end reads on an Illumina NovaSeq sequencer, yielding a median depth of approximately 40 million reads per sample. Molecular subtypes were predicted based on gene expression signatures by applying an Extreme Gradient Boosting (XGB) machine learning algorithm using the R package caret and trained on a previously classified reference cohort as well as using ALLCatchR (17).

# **7 References**

1. Brüggemann M, Droese J, Bolz I, Lüth P, Pott C, Neuhoff N von, et al. Improved assessment of minimal residual disease in B cell malignancies using fluorogenic consensus probes for real-time quantitative PCR. *Leukemia* 2000; **14**: 1419–1425.

2. Brüggemann M, Kotrova M. Minimal residual disease in adult ALL: Technical aspects and implications for correct clinical interpretation. *Hematology: the American Society of Hematology Education Program* 2017; **2017**: 13–21.

3. Brüggemann M, Kotrová M, Knecht H, Bartram J, Boudjogrha M, Bystry V, et al. Standardized next-generation sequencing of immunoglobulin and T-cell receptor gene recombinations for MRD marker identification in acute lymphoblastic leukaemia; a EuroClonality-NGS validation study. *Leukemia* 2019; **33**: 2241–2253.

4. Schilhabel A, Szczepanowski M, Gastel-Mol EJ, Schillalies J, Ray J, Kim D, et al. Patient specific real-time PCR in precision medicine – Validation of IG/TR based MRD assessment in lymphoid leukemia. *Frontiers in Oncology* 2022; **12**.

5. van der Velden VHJ, Cazzaniga G, Schrauder A, Hancock J, Bader P, Panzer-Grumayer ER, et al. Analysis of minimal residual disease by Ig/TCR gene rearrangements: Guidelines for interpretation of real-time quantitative PCR data. *Leukemia* 2007; **21**: 604–611.

6. van der Velden VHJ, Panzer-Grümayer ER, Cazzaniga G, Flohr T, Sutton R, Schrauder A, et al. Optimization of PCR-based minimal residual disease diagnostics for childhood acute lymphoblastic leukemia in a multi-center setting. *Leukemia* 2007; **21**: 706–713.

7. van der Velden VHJ, van Dongen JJM. MRD detection in acute lymphoblastic leukemia patients using Ig/TCR gene rearrangements as targets for real-time quantitative PCR. *Methods in molecular biology (Clifton, N.J.)* 2009; **538**: 115–150.

8. Kotrová M, Koopmann J, Trautmann H, Alakel N, Beck J, Nachtkamp K, et al. Prognostic value of low-level MRD in adult acute lymphoblastic leukemia detected by low- and high-throughput methods. *Blood advances* 2022; **6**: 3006–3010.

9. Schwartz S, Rieder H, Schläger B, Burmeister T, Fischer L, Thiel E. Expression of the human homologue of rat NG2 in adult acute lymphoblastic leukemia: Close association with MLL rearrangement and a CD10(-)/CD24(-)/CD65s(+)/CD15(+) B-cell phenotype. *Leukemia* 2003; **17**: 1589–1595.

10. Glier H, Heijnen I, Hauwel M, Dirks J, Quarroz S, Lehmann T, et al. Standardization of 8-color flow cytometry across different flow cytometer instruments: A feasibility study in clinical laboratories in Switzerland. *Journal of immunological methods* 2019; **475**: 112348.

11. Kalina T, Flores-Montero J, van der Velden VHJ, Martin-Ayuso M, Böttcher S, Ritgen M, et al. EuroFlow standardization of flow cytometer instrument settings and immunophenotyping protocols. *Leukemia* 2012; **26**: 1986–2010.

12. van Dongen JJM, Lhermitte L, Böttcher S, Almeida J, van der Velden VHJ, Flores-Montero J, et al. EuroFlow antibody panels for standardized n-dimensional flow cytometric immunophenotyping of normal, reactive and malignant leukocytes. *Leukemia* 2012; **26**: 1908–1975.

13. Glier H, Novakova M, Te Marvelde J, Bijkerk A, Morf D, Thurner D, et al. Comments on EuroFlow standard operating procedures for instrument setup and compensation for BD FACS Canto II, Navios and BD FACS Lyric instruments. *Journal of immunological methods* 2019; **475**: 112680.

14. Dworzak MN, Schumich A, Printz D, Pötschger U, Husak Z, Attarbaschi A, et al. CD20 up-regulation in pediatric B-cell precursor acute lymphoblastic leukemia during induction treatment: setting the stage for anti-CD20 directed immunotherapy. *Blood* 2008; **112**: 3982–3988.

15. Theunissen P, Mejstrikova E, Sedek L, van der Sluijs-Gelling AJ, Gaipa G, Bartels M, et al. Standardized flow cytometry for highly sensitive MRD measurements in B-cell acute lymphoblastic leukemia. *Blood* 2017; **129**: 347–357.

16. Bastian L, Hartmann AM, Beder T, Hänzelmann S, Kässens J, Bultmann M, et al. UBTF:ATXN7L3 gene fusion defines novel B cell precursor ALL subtype with CDX2 expression and need for intensified treatment. *Leukemia* 2022; **36**: 1676–1680.

17. Beder T, Hansen B-T, Hartmann AM, Zimmermann J, Amelunxen E, Wolgast N, et al. The Gene Expression Classifier ALLCatchR Identifies B-cell Precursor ALL Subtypes and Underlying Developmental Trajectories Across Age. *HemaSphere* 2023; **7**: e939.

18. Bastian L, Schroeder MP, Eckert C, Schlee C, Tanchez JO, Kämpf S, et al. PAX5 biallelic genomic alterations define a novel subgroup of B-cell precursor acute lymphoblastic leukemia. *Leukemia* 2019; **33**: 1895–1909.


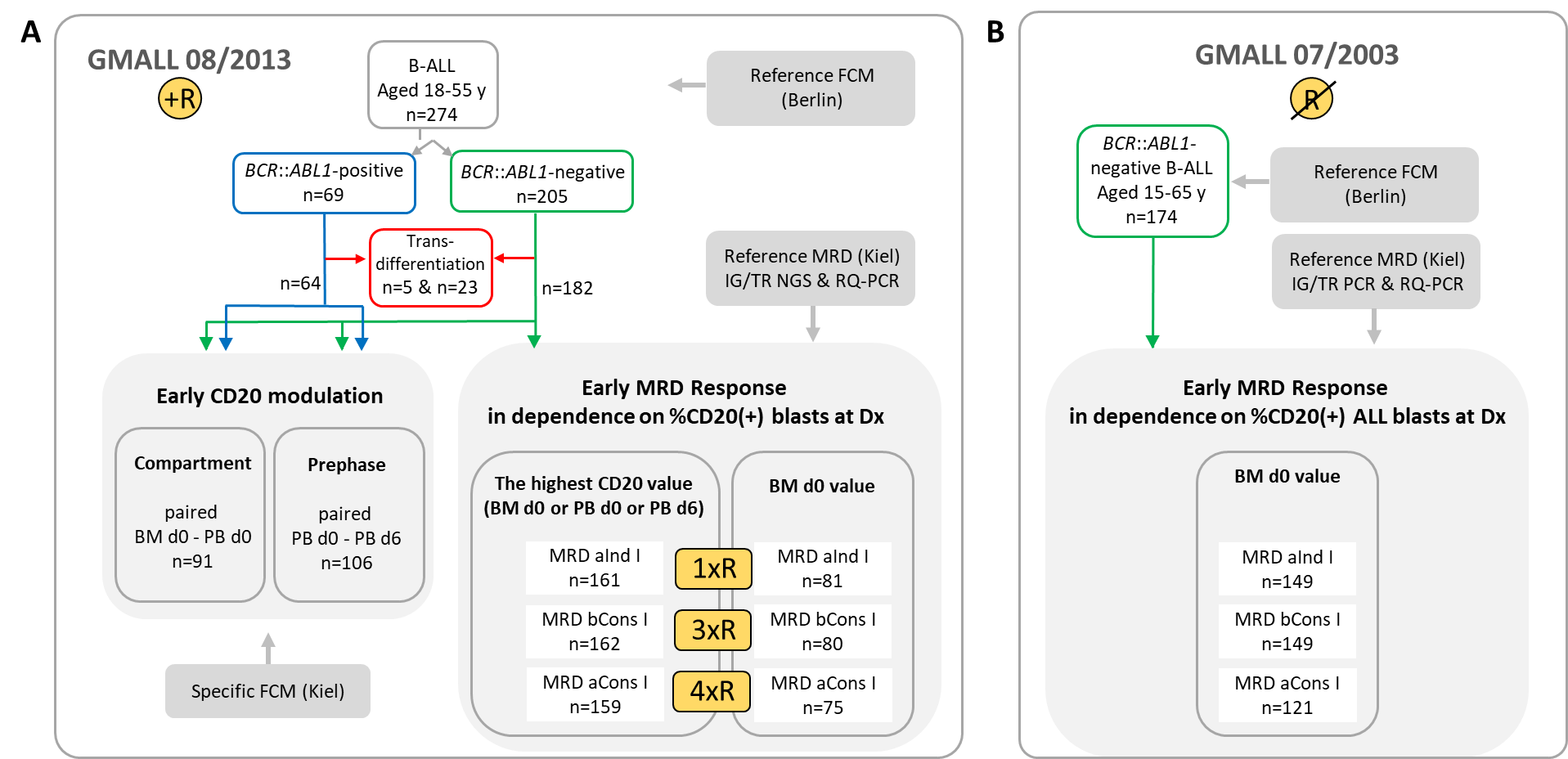


**Additional File 2**

**Supplementary Figure 1.** **Patient cohorts and analyses. (A)** In the GMALL 08/2013 trial, compartment-specific differences in baseline CD20 expression were investigated in bone marrow (BM) and peripheral blood (PB) of B-ALL patients (day 0, d0). Prephase-related CD20 expression modulation was monitored in PB d0 in comparison to PB d6 (day 6, end of prephase, before the first rituximab dose). Baseline leukemic CD20 expression (specifically, the percentage of CD20-positive blasts) was correlated with early response via IG/TR-based MRD values after Induction I (1x rituximab), before Consolidation I (3x rituximab), and after Consolidation I (4x rituximab) **(A)** within the GMALL 08/2013 trial and **(B)** within the GMALL 07/2003 trial before Amendment III in 2007 (without rituximab). Transdifferentiating cases (n=28) were excluded from further analyses due to immunophenotypic instability.


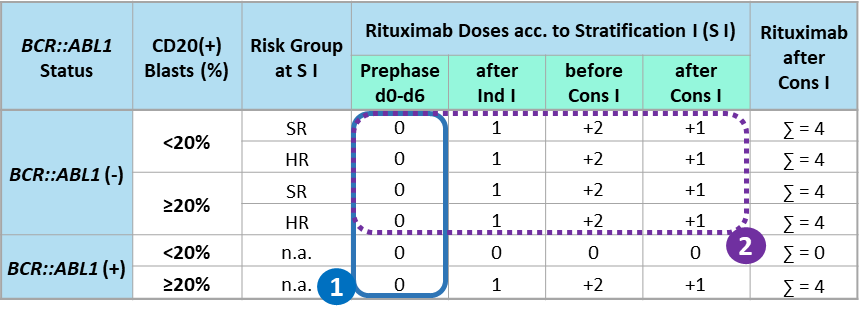


**Additional File 3**

**Supplementary Figure 2. Patient cohorts and analyses. (1) Early CD20 modulation:** In the GMALL 08/2013 trial, all newly diagnosed *BCR::ABL1*-negative and *BCR::ABL1*-positive B-ALL patients received the same cyclophosphamide- and dexamethasone-containing prephase regimen before induction chemotherapy and the first rituximab administration. CD20 expression was measured in all B-ALL patients at baseline [bone marrow (BM) and peripheral blood (PB) on day 0 (d0)] and in peripheral blood after prephase on day 6 (d6); **(2) Early MRD response:** CD20-positive and CD20-negative B-ALL patients without *BCR::ABL1* rearrangements uniformly received four rituximab doses early in treatment, starting at day 6 (start of Induction I), followed by two doses during Induction II and one at Consolidation I. These patients were eligible for a comprehensive analysis of early treatment response to rituximab after Induction I, before and after Consolidation I. In contrast, *BCR::ABL1*-positive B-ALL patients received rituximab only if they were diagnosed with CD20-positive B-ALL, and were excluded from the MRD analysis due to diverging disease biology and post-prephase treatment protocols.

**Additional File 4**

**Supplementary Table 1**: **List of antibodies used for multiparametric flow cytometry.** CD20 Antibody Panel; 1= 1^st^ version of antibody panel; EF= antibody panel according to EuroFlow Consortium protocol as described in Additional File 1: Materials and Methods.

| **Antigene** | **Clone** | **Fluorochrome** | **Manufacturer** | **CD20 Antibody Panel** |
| --- | --- | --- | --- | --- |
| CD3 | SK7 | PerCP-Cy5.5 | Becton Dickinson | 1 |
| CD9 | M-L13 | FITC | BD Pharmingen | 1 |
| CD10 | HI10a | BV510 | Becton Dickinson | 1 |
| CD10 | HI10a | PE | Becton Dickinson | 1 |
| CD10 | HI10a | APC | Becton Dickinson | EF |
| CD11a | HI111 | BV510 | BD Horizon | 1 |
| CD13 | L138 | PE | Becton Dickinson | 1 / EF |
| CD19 | J3.119 | PE-Cy7 | Beckman Coulter | 1 / EF |
| CD20 | 2H7 | PB | Biolegend | 1 / EF |
| CD20 | L27 | FITC | Becton Dickinson | 1 |
| CD22 | HIB22 | BV421 | BD Horizon | 1 |
| CD33 | P67.6 | PE | Becton Dickinson | 1 / EF |
| CD34 | 8G12 | PerCP-Cy5.5 | Becton Dickinson | 1 / EF |
| CD38 | HIT2 | PerCP-Cy5.5 | BD Pharmingen | 1 |
| CD38 | LS198-4-3 | APC-AF750 | Beckman Coulter | EF |
| CD45 | HI30 | PO | Invitrogen | EF |
| CD45 | 2D1 | APC-H7 | BD Pharmingen | 1 / EF |
| CD58 | AICD58.6 | APC | Beckman Coulter | 1 |
| CD66c | KOR-SA3544 | PE | Beckman Coulter | 1 / EF |
| CD71 | L01.1 | APC | Becton Dickinson | 1 / EF |
| CD73 | AD-2 | PE | BD Pharmingen | EF |
| CD81 | JS-81 | FITC | BD Pharmingen | EF |
| CD123 | AC145 | PE | Miltenyi | 1 / EF |
| CD304 | 12C2 | PE | Biolegend | EF |


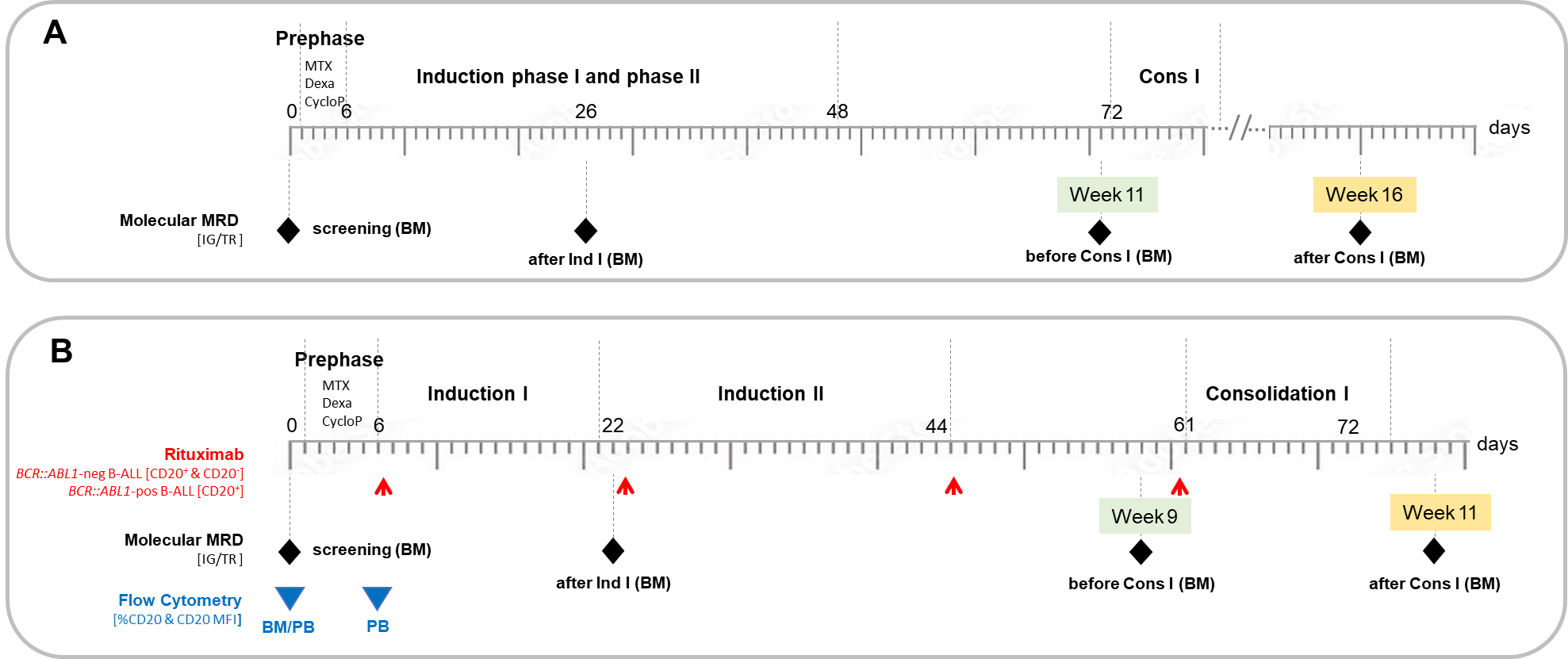


**Additional File 5**

**Supplementary Figure 3.** **GMALL 07/2003 and GMALL 08/2013 – Early Therapy Details and respective analyses.** **(A) GMALL 07/2003.** The patients analyzed were recruited before Amendment III in 2007 and did not receive rituximab. **(B) GMALL 08/2013.** Four doses of rituximab are uniformly administered until after Consolidation I in all *BCR*::*ABL1-*negative B-ALL cases, regardless of baseline CD20 expression (<20% CD20(+) blasts and ≥20% CD20(+) blasts). The multiparametric flow cytometry for CD20 assessment was performed at baseline in BM and/or PB, and at the end of prephase in PB on day 6 (before the first rituximab administration). In both trials, MRD was routinely monitored by clonotypic IG/TR rearrangements at the time points indicated on the time axis. Corresponding therapeutic time points between trials are uniformly colored. BM, bone marrow; IG/TR, immunoglobulin/T-cell receptor gene rearrangements as clonotypic molecular markers; MRD, measurable residual disease; PB, peripheral blood.


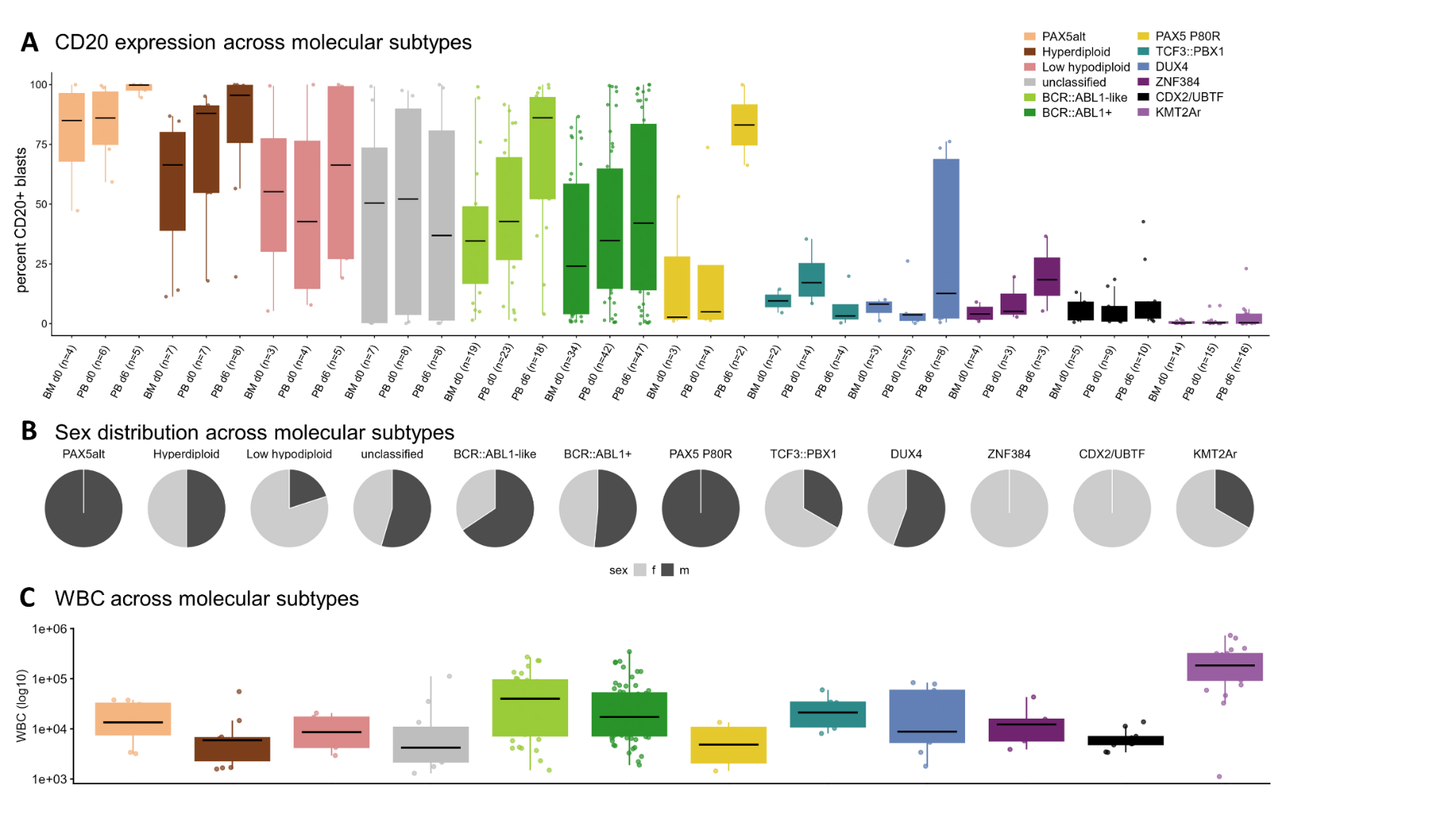


**Additional File 6**

**Supplementary Figure S4: CD20 expression dynamics across molecular subgroups of adult B-ALL. (A)** Distribution of CD20 expression in leukemic blasts at diagnosis in bone marrow (BM d0), diagnosis in peripheral blood (PB d0), and after prephase treatment in peripheral blood (PB d6), stratified by molecular subgroup. Individual patient values are shown as dots, with boxplots indicating median and interquartile range. Included are *BCR::ABL1+* patients and *BCR::ABL1* negative patients with RNA-seq, excluding subgroups with ≤3 patients (n = 187). Molecular subgroups are ordered according to decreasing CD20 expression **(B)** Sex distribution within each molecular subgroup. Blue and red sectors indicate male and female patients, respectively. **(C)** Distribution of white blood cell (WBC) counts at diagnosis across molecular subgroups. Individual patient values are shown together with boxplots indicating median and interquartile range.

**Supplementary Figure S4: CD20 expression dynamics across molecular subgroups of adult B-ALL. (A)** Distribution of CD20 expression in leukemic blasts at diagnosis in bone marrow (BM d0), diagnosis in peripheral blood (PB d0), and after prephase treatment in peripheral blood (PB d6), stratified by molecular subgroup. Individual patient values are shown as dots, with boxplots indicating median and interquartile range. Molecular subgroups are ordered according to decreasing CD20 expression and include only subgroups with at least 3 patients. **(B)** Sex distribution within each molecular subgroup. Blue and red sectors indicate male and female patients, respectively. **(C)** Distribution of white blood cell (WBC) counts at diagnosis across molecular subgroups. Individual patient values are shown together with boxplots indicating median and interquartile range.

**Additional File 7**

**Supplementary Table S2: Univariable and multivariable logistic regression for CD20-high status.** CD20-high was defined as CD20 expression ≥20% on leukemic blasts, and CD20-low as CD20 expression <20%. Odds ratios represent the odds of being CD20-high compared with CD20-low. The unclassified molecular subtype group was used as a reference because it showed a balanced distribution of CD20-high and CD20-low cases. Individual P values for molecular subtype rows compare each subtype with the unclassified reference group. The global P value for molecular subtype was calculated using a likelihood-ratio test and reflects the overall contribution of molecular subtype to the multivariable model. NE, not estimable. Included are *BCR::ABL1*+ patients and *BCR::ABL1* negative patients with RNA-seq (n = 198).

| **Variable** | **N** | **CD20 ≥20%**  **n (%)** | **CD20 <20%**  **n (%)** | **Univariable OR  (95% CI)** | **Univariable  P value** | **Multivariable OR  (95% CI)** | **Multivariable  P value** | **Multivariable  global P value** |
| --- | --- | --- | --- | --- | --- | --- | --- | --- |
| **Age** | 198 | 116 (58.6%) | 82 (41.4%) |  |  |  |  |  |
| Age, per 10 years | 198 | 116 (58.6%) | 82 (41.4%) | 0.86 (0.68–1.08) | 0.199 | 0.81 (0.58–1.11) | 0.192 |  |
| **Sex** | 198 | 116 (58.6%) | 82 (41.4%) |  |  |  |  |  |
| Female | 99 | 51 (51.5%) | 48 (48.5%) | Reference |  | Reference |  |  |
| Male | 99 | 65 (65.7%) | 34 (34.3%) | 1.80 (1.02–3.19) | ***0.044*** | 0.95 (0.44–2.06) | 0.896 |  |
| WBC | 198 | 116 (58.6%) | 82 (41.4%) |  |  |  |  |  |
| WBC, per 10,000/µL | 198 | 116 (58.6%) | 82 (41.4%) | 0.96 (0.92–0.99) | ***0.012*** | 0.99 (0.93–1.05) | 0.672 |  |
| **Molecular subtype** | 198 | 116 (58.6%) | 82 (41.4%) |  |  |  |  | ***<0.001*** |
| unclassified | 11 | 6 (54.5%) | 5 (45.5%) | Reference |  | Reference |  |  |
| BCR::ABL1-pos | 64 | 47 (73.4%) | 17 (26.6%) | 2.30 (0.62–8.54) | 0.212 | 3.00 (0.75–12.01) | 0.120 |  |
| BCR::ABL1-like | 32 | 26 (81.2%) | 6 (18.8%) | 3.61 (0.82–15.90) | 0.089 | 4.06 (0.88–18.80) | 0.073 |  |
| KMT2Ar | 21 | 1 (4.8%) | 20 (95.2%) | 0.04 (0.00–0.43) | ***0.008*** | 0.06 (0.00–0.75) | ***0.030*** |  |
| Hyperdiploid | 12 | 11 (91.7%) | 1 (8.3%) | 9.17 (0.86–97.70) | 0.066 | 7.97 (0.73–86.43) | 0.088 |  |
| DUX4 | 9 | 4 (44.4%) | 5 (55.6%) | 0.67 (0.11–3.92) | 0.654 | 0.59 (0.10–3.60) | 0.571 |  |
| PAX5alt | 8 | 8 (100.0%) | 0 (0.0%) | NE | NE | NE | NE |  |
| CDX2/UBTF | 10 | 2 (20.0%) | 8 (80.0%) | 0.21 (0.03–1.47) | 0.115 | 0.27 (0.03–2.11) | 0.212 |  |
| CEBP | 3 | 0 (0.0%) | 3 (100.0%) | NE | NE | NE | NE |  |
| ETV6::RUNX1 | 1 | 0 (0.0%) | 1 (100.0%) | NE | NE | NE | NE |  |
| HLF | 1 | 0 (0.0%) | 1 (100.0%) | NE | NE | NE | NE |  |
| IKZF1 N159Y | 3 | 0 (0.0%) | 3 (100.0%) | NE | NE | NE | NE |  |
| Low hypodiploid | 5 | 4 (80.0%) | 1 (20.0%) | 3.33 (0.28–40.29) | 0.344 | 3.60 (0.29–45.06) | 0.321 |  |
| MEF2D | 3 | 2 (66.7%) | 1 (33.3%) | 1.67 (0.11–24.26) | 0.708 | 1.69 (0.11–25.04) | 0.703 |  |
| PAX5 P80R | 4 | 2 (50.0%) | 2 (50.0%) | 0.83 (0.08–8.24) | 0.876 | 0.76 (0.07–8.04) | 0.822 |  |
| TCF3::PBX1 | 6 | 2 (33.3%) | 4 (66.7%) | 0.42 (0.05–3.31) | 0.407 | 0.45 (0.05–3.62) | 0.449 |  |
| ZNF384 | 5 | 1 (20.0%) | 4 (80.0%) | 0.21 (0.02–2.52) | 0.217 | 0.20 (0.02–2.58) | 0.219 |  |


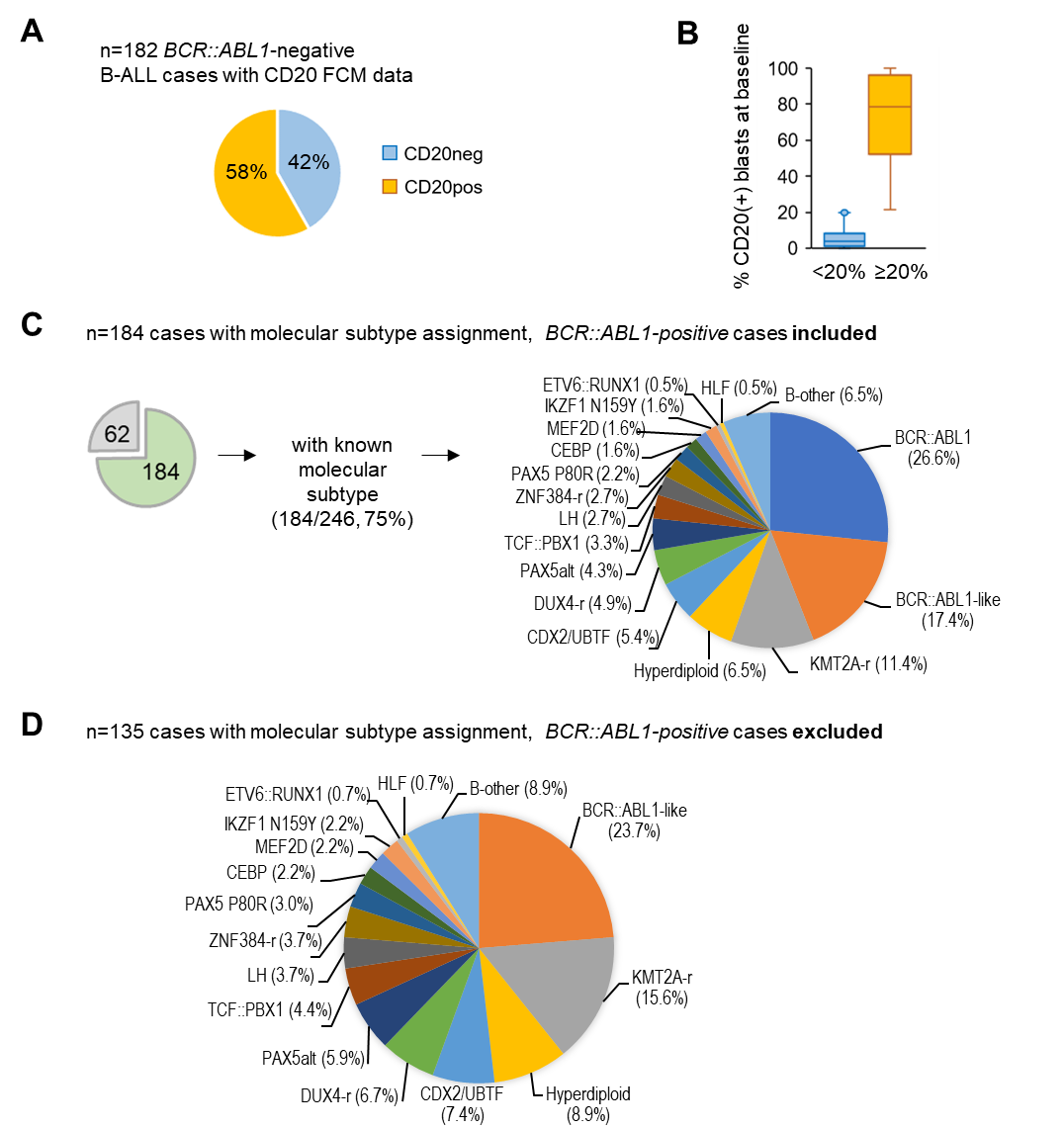


**Additional File 8**

**Supplementary Figure 5.** **CD20 expression and molecular subtypes within GMALL 08/2013.** **(A)** Based on initial CD20 expression, 182 *BCR::ABL1*-negative B-ALL cases from the GMALL 08/2013 cohort were classified as CD20-negative (76/182, 42%) or CD20-positive (106/182, 58%) B-ALL using the standard cutoff of ≥20% CD20(+) blasts at baseline; **(B)** The highest measured CD20-percentage value at baseline, derived either from bone marrow or peripheral blood at day 0 or from the peripheral blood after prephase (day 6) and before the first rituximab administration, is shown in CD20-negative (range 0.0-19.9%, median 3.8%) and CD20-positive (range 21.2-100.0%, median 78.6%) B-ALL cases; **(C)** Molecular-subtype assignment of 184 cases from the GMALL 08/2013 cohort (including *BCR::ABL1*-positive B-ALL): BCR::ABL1-like cases as the second frequent group, accounting for 17.4% of all cases; **(D)** Transcriptome-based molecular-subtype assignment of 135 *BCR::ABL1*-negative B-ALL cases from the GMALL 08/2013 cohort: BCR::ABL1-like cases account for 23.7% of the cases.
